# Shared genetic effect of kidney function on bipolar and major depressive disorders: a large-scale genome-wide cross-trait analysis

**DOI:** 10.1186/s40246-024-00627-3

**Published:** 2024-06-11

**Authors:** Simin Yu, Yifei Lin, Yong Yang, Xi Jin, Banghua Liao, Donghao Lu, Jin Huang

**Affiliations:** 1 Department of Urology, Innovation Institute for Integration of Medicine and Engineering, West China Hospital, Sichuan University, Chengdu, Sichuan People’s Republic of China; 2 Program in Genetic Epidemiology and Statistical Genetics, Department of Epidemiology, Harvard T.H. Chan School of Public Health, Boston, Massachusetts USA; 3 Health Management Center, General Practice Medical Center, Innovation Institute for Integration of Medicine and Engineering, West China Hospital, Sichuan University, Chengdu, Sichuan People’s Republic of China; 4 Department of Urology, Institute of Urology (Laboratory of Reconstructive Urology), West China Hospital, Sichuan University, Chengdu, Sichuan People’s Republic of China; 5 Institute of Environmental Medicine, Karolinska Institutet, Nobels Väg 13, 17177 Stockholm, Sweden; 6 Health Management Center, General Practice Medical Center and Medical Device Regulatory Research and Evaluation Centre, West China Hospital, Sichuan University, Chengdu, People’s Republic of China

**Keywords:** Genetic epidemiology, Cross-trait analysis, Kidney function, Bipolar disorder, Major depressive disorder

## Abstract

**Background:**

Epidemiological studies have revealed a significant association between impaired kidney function and certain mental disorders, particularly bipolar disorder (BIP) and major depressive disorder (MDD). However, the evidence regarding shared genetics and causality is limited due to residual confounding and reverse causation.

**Methods:**

In this study, we conducted a large-scale genome-wide cross-trait association study to investigate the genetic overlap between 5 kidney function biomarkers (eGFRcrea, eGFRcys, blood urea nitrogen (BUN), serum urate, and UACR) and 2 mental disorders (MDD, BIP). Summary-level data of European ancestry were extracted from UK Biobank, Chronic Kidney Disease Genetics Consortium, and Psychiatric Genomics Consortium.

**Results:**

Using LD score regression, we found moderate but significant genetic correlations between kidney function biomarker traits on BIP and MDD. Cross-trait meta-analysis identified 1 to 19 independent significant loci that were found shared among 10 pairs of 5 kidney function biomarkers traits and 2 mental disorders. Among them, 3 novel genes: *SUFU*, *IBSP*, and *PTPRJ*, were also identified in transcriptome-wide association study analysis (TWAS), most of which were observed in the nervous and digestive systems (FDR < 0.05). Pathway analysis showed the immune system could play a role between kidney function biomarkers and mental disorders. Bidirectional mendelian randomization analysis suggested a potential causal relationship of kidney function biomarkers on BIP and MDD.

**Conclusions:**

In conclusion, the study demonstrated that both BIP and MDD shared genetic architecture with kidney function biomarkers, providing new insights into their genetic architectures and suggesting that larger GWASs are warranted.

**Supplementary Information:**

The online version contains supplementary material available at 10.1186/s40246-024-00627-3.

## Introduction

Considerable evidence has suggested a notable correlation between bipolar disorder (BIP) and major depressive disorder (MDD) [1–4]. Both mental disorders share common features such as major depressive episodes. Notably, genetic epidemiological [5] and genome-wide linkage studies [6] support the notion that there is an overlap in genetic risk factors between both disorders. Accumulating studies showed that both BIP and MDD had significant association with impaired kidney function [7–10]. For example, Clinical observations have indicated that depression was a risk factor for progression of chronic kidney disease (CKD) [11, 12]. Additionally, the decline of estimated glomerular filtration rate (eGFR) was discovered in patients with BIP in a population from northern Sweden [13]. Another nationwide population-based study demonstrated that patients with BIP had increased more than 2 folds of incidence of CKD [14].

Conversely, patients with CKD were up to 3 times more likely to be hospitalized for mental disorders, especially depression, compared to patients with other chronic illnesses including cardiovascular diseases and gastrointestinal diseases [15]. Moreover, impaired kidney function biomarkers including declined BUN and eGFRcrea are also considered to be independent risk factors for mental disorders [16, 17]. A cross-sectional investigation conducted in the United Kingdom revealed a higher prevalence of CKD among individuals with mental disorders compared to the general population (*P* < 0.05, n = 4295)[18]. Mental disorders such as major depression also has the potential to affect the CKD patients’ ability to make decisions and to understand the complex treatment, such as fluid and dietary restrictions [19]. Prior research has also unveiled the underdiagnosis and undertreatment of depression among patients undergoing hemodialysis for end-stage renal disease [20–22]. Despite these findings, the underlying mechanism linking impaired kidney function biomarkers and mental disorders remains unclear, presenting a significant challenge in the diagnosis and treatment of mental disorders.

Genome-wide association studies (GWAS) have unveiled numerous genetic variants associated with kidney function biomarkers and mental disorders [23–27]. A recently published mendelian randomization study showed kidney damage had a causal effect on cerebral cortex [28], indicating a shared genetic architecture between impaired kidney function and mental disorders. However, it remains unclear whether the overall genetic correlation between these 2 types of diseases would be attributed to a few loci across the genome.

Therefore, we conducted a large-scale genome-wide cross-trait association study with ~ 1,000,000 individuals of European ancestry to investigate the genetic overlap between 5 kidney function biomarkers (creatinine-based estimated glomerular filtration rate (eGFRcrea), cystatin C-based estimated glomerular filtration rate (eGFRcys), blood urea nitrogen (BUN), serum urate, and urinary albumin-to-creatinine ratio (UACR)) and 2 mental disorders (BIP, MDD). Using both linkage disequilibrium (LD) score regression and bidirectional Mendelian randomization (MR), we aimed to explore the genetic correlation and causal relationship between the 2 sets of traits. Furthermore, we conducted a genome-wide cross-trait meta-analysis with GWAS summary statistics to identify shared genetic loci and provide insights into the molecular mechanisms underlying their shared genetic liability and potential causal relationship.

## Methods and material

### Study design and data sources

**Fig. 1:**
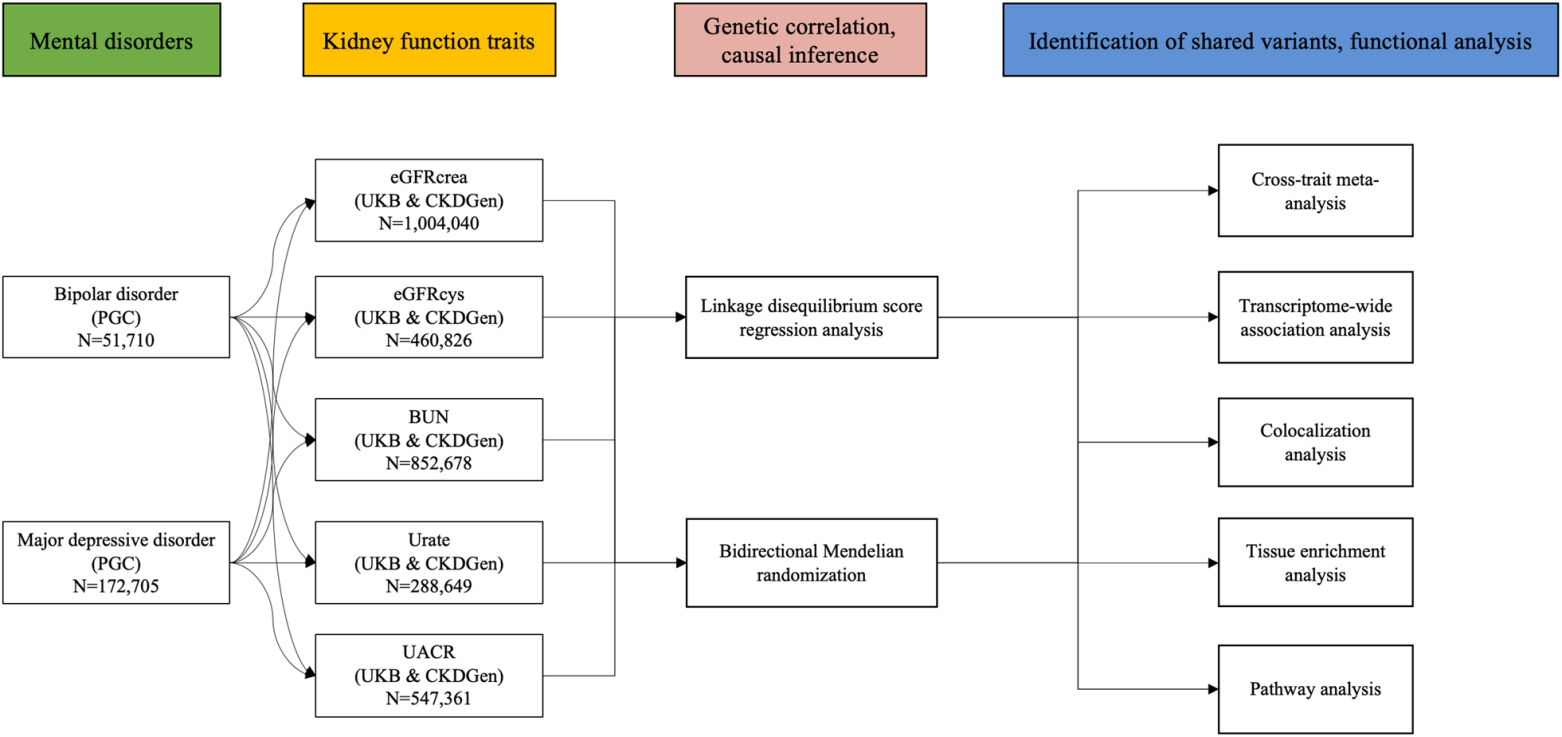
Overall study design. PGC: Psychiatric Genomics Consortium; UKB: UK Biobank; CKDGen: Chronic Kidney Disease Genetics Consortium; BIP: bipolar disorder; MDD: major depressive disorder; BUN: blood urea nitrogen; eGFRcys: cystatin C-based estimated glomerular filtration rate; UACR: urine albumin-creatinine ratio; eGFRcrea: creatinine-based estimated glomerular filtration rate

The workflow of our analysis was shown in Fig. 1. There were 4 main parts in our study: genetic correlation, causal inference, identification of shared variants and functional analysis between 5 kidney function biomarker traits and 2 mental disorders.

To minimize population stratification bias, we included individuals of European ancestry in our analyses. We obtained summary statistics from publicly available GWASs. Regarding kidney function biomarker traits, all the summary statistics were based on continuous biomarkers, including eGFRcrea (N = 1,004,040), eGFRcys (N = 460,826), BUN (N = 852,678), urate (N = 288,649), and UACR (N = 547,361), from the Chronic Kidney Disease Genetics Consortium and UK Biobank. For mental disorder traits, we retrieved summary statistics from publicly available GWAS studies of the Psychiatric Genomics Consortium (PGC): BIP (N = 51,710), and MDD (N = 172,705). Our analysis relied solely on publicly available summary-level data, for which all subjects provided informed consent in accordance with the original GWAS protocols. Furthermore, the original GWAS authors obtained all necessary ethics approvals for their studies. Additional information regarding each dataset can be found in Additional file 1: Supplementary Table 1.

### Linkage disequilibrium score regression (LDSC)

To examine the genetic correlation of kidney function biomarkers on BIP and MDD, we utilized the LDSC software to conduct a genome-wide post-GWAS genetic correlation analysis(Bulik-Sullivan et al., 2015; “LD Score regression distinguishes confounding from polygenicity in genome-wide association studies | Nature Genetics,” n.d.) [29, 30]. LDSC estimates the genetic correlation between two traits based on the true causal effects (ranging from − 1 to 1). In this analysis, the GWAS effect size estimate was calculated for each SNP. SNPs in high-linkage disequilibrium regions show higher test statistics compared with SNPs in low-linkage disequilibrium regions. LDSC also provides a self-estimated intercept to indicate the overlapped between studies. To account for multiple testing, we applied the false discovery rate (FDR) Benjamini–Hochberg method considering a P-value of 0.01 (0.05/5) as the significance threshold for LD score regression analysis [31].

### Multi-Trait Analysis of GWAS (MTAG)

After evaluating the genetic correlations among all traits, we applied MTAG to identify novel loci with strong signals for kidney function biomarker trasits and mental disorders and to detect shared genetic variants between the two traits. MTAG is a statistical method that is used to increase power and detect novel loci in GWAS studies [32]. It combines summary statistics from multiple traits while taking into account the correlation between them, then executes a meta-analysis to recognize significant genetic loci. The MTAG estimator is an extension of inverse-variance-weighted meta-analysis, which obtains trait-specific association statistics by using summary statistics from single-trait GWAS. Moreover, MTAG association statistics provide additional power and minimal inflation of the FDR for each trait examined with high correlation, aligning with theoretical expectations [32].

### Colocalization analysis

The colocalization analysis is a statistical method used to assess the overlap of association loci between two different GWAS studies by Bayesian analyses [33]. The method aims to ascertain whether the available data substantiate the presence of a shared causal variant influencing both traits. In instances where a locus exhibits multiple independent associations with a given trait, the algorithms selectively prioritize the most robust among these distinct association signals [33]. In our study, we used the “coloc” R package to conduct genetic colocalization analysis between kidney function biomarker traits and mental disorder traits. We calculated the probability that each locus is a shared genetic causal variant (PPH4), with loci having posterior probabilities greater than 0.5 considered colocalized. To provide additional information on the genes involved, we used genome browser annotation to connect the loci with their nearest gene.

### Transcriptome-wide association study analysis (TWAS) and Tissue‐specific expression analysis

To investigate the correlation between kidney function and mental disorders with regard to transcriptome gene expression in specific tissues, we performed a TWAS using FUSION software (version 1.4.1) package based on 49 post-mortem tissues expression weights from the Genotype-Tissue Expression project (GTEx, version8)[34, 35]. To mitigate the risk of false positives, we applied the FDR Benjamini–Hochberg procedure (FDR < 0.05) to correct all gene-tissue pairs based on TWAS *P*-values [31].

To explore the tissue specificity of the phenotype identified by our study, we conducted an SNP-based tissue enrichment analysis using the Functional Mapping and Annotation (FUMA) software [36]. FUMA uses gene-property analyses to test the associations between tissue-specific gene expression profiles in general GTEx V8 tissues and GWAS hits. Furthermore, pathway enrichment analyses of a set of genes were also conducted using FUMA, involving the Gene Ontology (GO) project and Kyoto Encyclopedia of Genes and Genomes (KEGG). The GO project consists of three fundamental domains: biological process (BP), cellular component (CC), and molecular function (MF), while the KEGG is a valuable database for exploring genomes, biological pathways, diseases, chemicals, and drugs.

### Mendelian randomization analysis

For forward MR, instrumental variables (IV) for two-sample MR were identified as genetic variants associated with BIP and MDD. In the reverse MR, IVs were chosen as genetic variants associated with the above mentioned 5 kidney function traits. To ensure independence of the instruments, all genetic variants were clumped using PLINK (–clump-p1 5e-8 –clump-p2 1e-5 –clump-r2 0.1 –clump-kb 1000).

We performed several MR methods to investigate potential causal associations of kidney function biomarker traits on BIP and MDD. The inverse-variance weighted (IVW) method was employed as the primary analysis to ensure a precise estimate of the summary-level data [37]. However, the performance of the IVW method is prone to be influenced by invalid instrumental variables. Therefore, we first conducted MR-Egger regression to account for unmeasured pleiotropy, as this method is sensitive to outliers and provides consistent estimates of the causal effect [38]. Moreover, we employed the mode-based estimate (MBE) and contamination mixture (ConMix) methods to mitigate the effects of weak instrument bias, pleiotropy, and extreme outliers which helped validate the relationship [39, 40]. Given that the different MR methods rely on different assumptions for valid inferences, we can expect to obtain reliable MR results.

Generally, all the analyses were conducted using R software 4.0.3. The IVW, MR–Egger, MBE and ConMix methods were performed using the “MendelianRandomization” package.

## Results

### Genome-wide genetic correlation of kidney function biomarker traits on BIP and MDD

We investigated the genetic correlations between mental disorders and kidney function biomarker traits using LDSC (Table 1, Additional file 2: Supplementary Table 2). We observed moderate but positive genetic correlations (R_g_) between BIP and eGFRcrea (R_g_ = 0.079, FDR = 2.75 × 10^–3^), eGFRcys (R_g_ = 0.078, FDR = 2.75 × 10^–3^), and UACR (R_g_ = 0.098, FDR = 3.17 × 10^–3^). We also observed a negative genetic correlation (R_g_) between MDD and eGFRcys (R_g_ = -0.099, FDR = 3.13 × 10^–4^).

**Table 1:** Genome-wide genetic correlation kidney function biomarker traits on BIP and MDD

Mental disorders	Kidney function biomarkers	Rg	Rg_SE	Zs	P	FDR
BIP	BUN	− 0.006	0.027	− 0.231	8.18E−01	8.18E−01
	eGFRcrea	0.079	0.024	3.252	1.10E−03	2.75E−03
	eGFRcys	0.078	0.023	3.429	6.00E−04	2.75E−03
	UACR	0.098	0.032	3.100	1.90E−03	3.17E−03
	urate	− 0.035	0.023	− 1.520	1.29E−01	1.61E−01
MDD	BUN	− 0.017	0.023	− 0.746	4.56E−01	5.70E−01
	eGFRcrea	0.020	0.024	0.845	3.98E−01	5.70E−01
	eGFRcys	− 0.099	0.025	− 4.003	6.26E−05	3.13E−04
	UACR	− 0.030	0.028	− 1.060	2.89E−01	5.70E−01
	urate	0.008	0.035	0.232	8.17E−01	8.17E−01

Rg: genetic correlation estimate; SE: standard error of genetic correlation estimate; BIP: bipolar disorder; MDD: major depressive disorder; BUN: Blood Urea Nitrogen; eGFRcys: cystatin C-based estimated glomerular filtration rate; UACR: Urine Albumin-Creatinine Ratio; eGFRcrea: creatinine-based estimated glomerular filtration rate

### Cross‑trait *meta*‑analysis of kidney function biomarker traits on BIP and MDD

We applied MTAG for genome-wide cross-trait meta-analysis to identify genetic loci associated with kidney function biomarker traits and mental disorders. Shared genetic loci were defined by selecting SNPs with cross-trait meta-analysis *P*_mtag_ < 5 × 10^–8^ and single trait *P* < 5 × 10^–4^.

We identified a total of 69 independent loci that were shared among 10 pairs of 5 kidney function biomarkers and 2 mental disorders. For BIP, 5 significant shared loci were identified associated with BUN, 19 significant shared loci were identified associated with eGFRcrea, 13 significant shared loci were identified associated with eGFRcys, 6 significant shared loci were identified associated with UACR, 4 significant loci were identified associated with urate. For MDD, 3 significant shared loci were identified associated with BUN, 8 significant shared loci were identified associated with eGFRcrea, 11 significant loci were identified associated with eGFRcys, 1 significant locus were identified associated with UACR (PMDD = 4.98 × 10–3, PUACR = 8.90 × 10–10, Pmtag = 1.22 × 10–8), 6 significant loci were identified associated with urate (Table 2). The Manhattan plots of MTAG results were listed in Supplementary Figs. 1–10.

**Table 2:** TOP SNP of each 10 pairs of kidney function biomarker traits and mental disorder traits in multi-trait analysis of GWAS (P_mtag < 5e−8, single trait P < 5e−4)

MTAG trait pair		TOP significant SNP	CHR	Position	Kidney function biomarker		Mental disorder		P_mtag	Gene category	Gene/nearest Gene
Mental disorders	Kidney function biomarker traits				Beta	P	Beta	P			
BIP	BUN	rs2070803	1	1.55E+08	1.04E−02	3.08E−99	− 4.84E−02	4.36E− 04	1.63E−82	Intronic	MUC1
		rs1260326	2	27,730,940	5.30E−03	1.01E−26	6.34E−02	3.18E−06	1.44E−30	Exonic	GCKR
		rs35897939	8	1.28E+08	4.80E−03	2.49E−07	9.66E−02	1.58E−04	4.60E−10	ncRNA_intronic	PCAT1
		rs795232	11	30,773,321	− 5.00E−03	1.23E−08	− 8.75E−02	4.37E−04	3.06E−10	Intergenic	MPPED2-AS1
		rs72810571	2	28,115,910	7.60E−03	1.64E−07	1.33E−01	4.42E−04	4.97E−10	Intronic	BABAM2
	eGFRcrea	rs11697103	20	14,688,818	− 1.90E−03	4.16E−09	7.78E−02	6.15E−07	1.43E−77	Intronic	MACROD2
		rs1260326	2	27,730,940	5.10E−03	1.84E−74	6.34E−02	3.18E−06	2.75E−08	Exonic	GCKR
		rs41499647	3	50,525,154	− 1.80E−03	3.13E−07	− 7.90E−02	3.83E−06	1.43E−77	Intronic	CACNA2D2
		rs6059908	20	33,132,159	2.50E−03	2.78E−13	− 7.95E−02	4.74E−06	5.43 E−13	Intergenic	DYNLRB1
		rs3118119	1	1.5E+08	2.70E−03	4.76E−13	− 8.51E−02	4.99E−06	1.42 E−14	Intergenic	AC242988.2
		rs36134929	6	50,885,608	− 1.90E−03	2.58E−08	− 7.01E−02	6.73E−06	1.46 E−09	Intergenic	TFAP2B
		rs7307046	12	48,178,194	− 1.60E−03	1.21E−08	− 5.65E−02	3.88E−05	3.24 E−14	Intronic	HDAC7
		rs12912342	15	85,248,216	− 2.00 E−03	4.19 E−12	5.81 E−02	4.09 E−05	1.46 E−10	Intronic	SEC11A
		rs13083728	3	52,868,445	2.50 E−03	4.20 E−15	− 6.43 E−02	5.42 E−05	1.17 E−09	Intronic	AC006254.1
		rs3787433	20	60,833,870	1.60 E−03	1.02 E−08	5.31 E−02	7.52 E−05	4.86 E−15	Intronic	OSBPL2
		rs17391694	1	78,623,626	− 1.90 E−03	2.65 E−06	− 9.05 E−02	8.59 E−05	1.97 E−10	Intergenic	GIPC2
		rs5994431	22	32,210,300	− 1.40 E−03	2.02 E−05	− 6.00 E−02	1.23 E−04	8.71 E−09	Intronic	DEPDC5
		rs74873433	2	28,277,414	− 3.10 E−03	8.65 E−08	− 1.07 E−01	1.61 E−04	1.17E−08	Intronic	BABAM2
		rs62141290	2	27,783,392	3.40E−03	1.98E−24	6.20E−02	2.58E−04	6.89E−10	Intronic	C2orf16
		rs6601411	8	9,892,299	1.40E−03	7.04E−05	6.06E−02	2.74E−04	4.65E−36	ncRNA_intronic	AC034111.2
		rs4614892	2	1.82E+08	1.40E−03	4.82E−06	5.43E−02	3.80E−04	2.00E−08	Intergenic	SCHLAP1
		rs141428740	2	27,735,033	3.40E−03	3.03E−08	1.05E−01	4.09E−04	2.31E−08	Intronic	GCKR
		rs148696809	6	28,934,352	3.20E−03	1.68E−12	8.21E−02	4.10E−04	3.05E−11	Intergenic	KRT18P1
		rs6785570	3	1.42E + 08	− 1.70E−03	1.01E−09	− 4.92E−02	4.43E−04	1.36E−19	Intronic	ATP1B3
	eGFRcys	rs2388334	6	98,591,622	− 2.00E−03	1.15E−05	− 7.13E−02	8.62E−08	3.12E−09	ncRNA_intronic	AL589740.1
		rs174568	11	61,593,816	2.80E−03	8.04E−09	7.16E−02	3.89E−07	2.97E−14	Exonic	FADS2
		rs1260326	2	27,730,940	4.00E−03	2.81E−17	6.34E−02	3.18E−06	4.83E−24	Exonic	GCKR
		rs10014468	4	1.24E+08	− 3.90E−03	1.12E−04	− 1.28E−01	1.01E−05	2.96E−08	Intergenic	IL2
		rs34517439	1	78,450,517	− 6.50E−03	7.00E−20	− 9.42E−02	7.56E−05	1.02E−29	Intronic	DNAJB4
		rs34676049	6	28,453,618	5.80E−03	2.05E−15	9.35E−02	7.93E−05	1.59E−24	ncRNA_intronic	Z98745.2
		rs6714697	2	1.98E+08	− 3.10E−03	4.78E−10	− 5.58E−02	1.11E−04	2.52E−15	Intronic	RFTN2
		rs867282	2	27,951,658	− 2.90E−03	1.69E−07	− 5.89E−02	1.81E−04	1.02E−13	Intergenic	LINC01460
		rs59322673	12	1.11E+08	− 3.40E−03	7.28E−13	− 5.09E−02	2.03E−04	1.52E−17	Intergenic	AC144522
		rs1918863	2	73,644,481	− 4.90E−03	4.99E−19	− 5.86E−02	2.04E−04	3.26E−32	Intronic	ALMS1
		rs138124594	3	37,206,515	2.20E−03	2.35E−06	5.09E−02	2.35E−04	3.99E−09	Intronic	LRRFIP2
		rs35120058	6	27,358,166	5.30E−03	6.77E−13	8.53E−02	3.10E−04	9.38E−21	Intronic	ZNF391
		rs11542821	16	67,984,589	− 5.00E−03	2.07E−06	− 1.06E−01	4.11E−04	8.14E−11	Exonic	SLC12A4
	UACR	rs4665972	2	27,598,097	1.82E−02	1.17E−18	6.85E−02	7.50E−07	5.22E−22	Intronic	SNX17
		rs215001	6	1.53E+08	9.90E−03	1.53E−06	6.31E−02	5.12E−06	1.84E−09	Intronic	SYNE1
		rs62533709	9	37,003,704	2.56E−02	0.0022	9.59E−02	6.08E−06	6.36E−09	ncRNA_Intronic	AL161781.2
		rs2165446	1	1.64E+08	9.50E−03	1.40E−06	5.41E−02	5.14E−05	3.60E−09	Intergenic	NUF2
		rs77990632	8	42,609,001	− 2.00E−02	4.60E−06	− 1.26E−01	1.38E−04	1.87E−08	Intronic	CHRNA6
		rs2075571	1	1.55E+08	1.61E−02	5.36E−16	4.94E−02	3.37E−04	9.96E−19	ncRNA_Intronic	AC234582.1
	urate	rs7629072	3	52,305,633	− 1.72E−02	1.98E−08	− 6.55E−02	1.18E−06	7.69E−09	Intronic	WDR82
		rs1260326	2	27,730,940	6.60E−02	2.97E−95	6.34E−02	3.18E−06	2.67E−49	Exonic	GCKR
		rs12892189	14	1.04E+08	1.73E−02	4.94E−07	5.77E−02	4.95E−05	4.43E−09	ncRNA_intronic	LINC00637
		rs2070803	1	1.55E+08	5.42E−02	2.12E−60	− 4.84E−02	4.36E−04	8.79E−31	Intronic	MUC1
MDD	BUN	rs7893954	10	1.04E+08	2.90E−03	1.84E−07	3.59E−02	4.04E−05	1.57E−12	Intronic	SUFU
		rs4714291	6	40,003,632	− 2.20E−03	7.30E−05	− 3.58E−02	5.08E−05	6.96E−09	Intergenic	MOCS1
		rs76400970	11	46,231,891	3.60E−03	1.38E−05	4.45E−02	2.62E−04	9.11E−09	Intergenic	AC024475.4
	eGFRcrea	rs148696809	6	28,934,352	3.20E−03	1.68E−12	7.73E−02	4.36E−09	1.36E−19	Intergenic	KRT18P1
		rs35984974	6	27,410,422	− 3.20E−03	4.58E−13	− 7.63E−02	5.11E−09	1.66E−19	Intergenic	AL031118.1
		rs9393715	6	26,375,645	− 3.10E−03	2.26E−13	− 6.91E−02	1.93E−08	6.21E−18	Intronic	BTN3A2
		rs2523593	6	31,326,703	3.90E−03	6.54E−21	5.62E−02	3.34E−06	1.52E−26	Intergenic	HLA-B
		rs1008438	6	31,783,208	2.60E−03	7.11E−20	3.52E−02	1.53E−05	1.20E−20	Upstream	HSPA1A
		rs3828906	6	31,464,918	− 1.60E−03	2.98E−07	− 3.48E−02	6.84E−05	4.04E−08	Intronic	MICB
		rs2807852	1	2.21E+08	1.60E−03	3.95E−08	3.03E−02	2.29E−04	2.39E−08	ncRNA_intronic	HLX-AS1
		rs665058	11	57,579,166	− 2.00E−03	4.54E−12	2.77E−02	4.84E−04	6.94E−13	Intronic	CTNND1
	eGFRcys	rs144447022	6	29,244,219	− 5.80E−03	4.24E−16	− 7.87E−02	2.23E−09	1.52E−29	Intergenic	OR2J2
		rs71537572	6	27,970,715	5.60E−03	4.99E−14	7.48E−02	2.24E−08	3.05E−27	Intergenic	OR2B6
		rs57440165	6	26,843,517	5.10E−03	8.68E−12	7.60E−02	2.52E−08	5.87E−24	Intergenic	AL513548.1
		rs2442722	6	31,320,241	− 6.70E−03	7.84E−31	− 5.30E−02	2.84E−07	3.14E−43	Intronic	HLA-B
		rs2233960	6	31,081,052	− 3.00E−03	8.22E−09	− 4.04E−02	7.74E−06	1.64E−16	Upstream	C6orf15
		rs1008438	6	31,783,208	3.70E−03	2.85E−15	3.52E−02	1.53E−05	8.09E−22	Upstream	HSPA1A
		rs139747027	6	30,216,186	2.10E−03	0.00104	4.59E−02	2.05E−05	2.09E−08	ncRNA_intronic	HCG17
		rs2859368	6	28,418,039	2.90E−03	2.44E−10	3.23E−02	6.62E−05	5.77E−15	intergenic	ZSCAN23
		rs13200921	6	25,790,378	4.00E−03	7.31E−08	5.13E−02	1.55E−04	2.61E−14	intronic	SLC17A1
		rs487762	6	33,566,158	2.10E−03	7.41E−06	2.78E−02	4.51E−04	2.45E−09	intergenic	LINC00336
		rs56263644	2	86,671,074	− 2.60E−03	3.95E−04	− 4.17E−02	4.87E−04	4.95E−08	intronic	KDM3A
	UACR*	rs17035646	1	10,796,547	1.28E−02	8.90E−10	2.41E−02	4.98E−03	1.22E−08	intronic	CASZ1
	urate	rs7531118	1	72,837,239	− 9.10E−03	0.00844	− 4.50E−02	2.15E−08	2.03E−09	ncRNA_intronic	AL513166.2
		rs115557272	4	88,731,092	− 3.35E−02	1.22E−04	− 8.60E−02	6.01E−06	4.41E−11	Intronic	IBSP
		rs10086261	8	76,521,095	2.31E−02	2.79E−09	3.84E−02	5.09E−05	3.81E−10	Intergenic	HNF4G
		rs1468662	14	73,283,742	2.51E−02	4.00E−05	6.93E−02	8.55E−05	3.53E−09	Intronic	DPF3
		rs4665390	2	27,989,909	− 1.68E−02	6.01E−07	− 3.56E−02	8.79E−05	2.09E−11	Intergenic	LINC01460
		rs76088737	11	48,071,717	2.78E−02	1.88E−04	6.28E−02	2.29E−04	2.91E−08	Intronic	PTPRJ

^*^For MTAG between UACR and MDD, we did not find any locus under the threshold of Pmtag < 5E-8, and single trait P < 5E-4. Here we display the most significant locus between UACR and MDD, which is rs17035646 (P_UACR_ = 8.90E-10, P_MDD_ = 4.98E-03, Pmtag = 1.22E-08)

In the meta-analysis of BIP with 5 kidney function biomarker traits, the strongest association signals were localized to the *MUC1*(index SNP: rs2070803, P_mtag_ = 1.63 × 10^–82^) for BUN, *MACROD2* (index SNP: rs11697103, P_mtag_ = 1.43 × 10^–77^) for eGFRcrea, *AL589740.1* (index SNP: rs2388334, P_mtag_ = 3.26 × 10^–32^) for eGFRcys, *SNX17* (index SNP: rs4665972, P_mtag_ = 3.26 × 10^–32^) for UACR, and *WDR82*(index SNP: rs7629072, P_mtag_ = 2.67 × 10^–49^) for urate. For the meta-analysis of MDD with 5 kidney function biomarker traits, the strongest association signals were localized to the *SUFU* (index SNP: rs7893954, P_mtag_ = 1.57 × 10^–12^) for BUN, *HLA*-*B (*index SNP: rs2523593, P_mtag_ = 1.52 × 10^–26^) for eGFRcrea, *HLA-B* (index SNP: rs2442722, P_mtag_ = 3.15 × 10^–43^) for eGFRcys, *CASZ1* (index SNP: rs17035646, P_mtag_ = 1.22 × 10^–8^) for UACR, *LINC01460* (index SNP: rs4665390, P_mtag_ = 2.09 × 10^–11^) for urate.

Among the 69 loci, we would highlight 4 overlapped loci across 10 pairs of all the traits (Additional file 3: Supplementary Table 3). Notably, the first which also the only locus (index SNP: rs148696809, Pmtag = 2.09 × 10^–11^) shared by eGFRcrea with both BIP (P = 4.10 × 10^–4^) and MDD (P = 4.36 × 10^–9^) was in proximity to *KRT18P1*, which is a pseudogene of keratin [40]. Another 3 loci were shared by kidney function biomarker traits. The second locus (index SNP: rs1260326, Pmtag = 3.18 × 10^–6^) was shared by 4 kidney function biomarkers (BUN (P = 1.01 × 10^–26^), eGFRcrea (P = 1.84 × 10^–74^), eGFRcys (P = 2.81 × 10^–17^), urate (P = 2.97 × 10^–95^)) with BIP. SNP rs1260326 was mapped to *GCKR* gene, which typically suppresses GCK function by binding to GCK in the fast phase [41–43]. The third locus (index SNP: rs2070803, Pmtag = 1.63 × 10^–82^) was common to BUN (P = 3.08 × 10^–99^) and urate (P = 2.12 × 10^–60^). SNP rs2070803 was mapped to the genes *MUC1*, which play roles in cell surface protein coding and serum uric acid levels [44, 45]. The fourth locus (index SNP: rs1008438, Pmtag = 1.53 × 10^–5^) shared by MDD with eGFRcrea(P = 7.11 × 10^–20^) and eGFRcys(P = 2.85 × 10^–15^) was mapped to the genes *HSPA1A* and *HSPA1L*, which encode for heat shock protein family A members [46, 47].

### Colocalization

We further conducted colocalization analysis to investigate the causal shared genetic variants between 5 kidney function biomarker traits and 2 mental disorder traits. The colocalization analysis showed that BUN shared a causal variant (rs9290867) with MDD and BIP (PPH4 > 0.5). Additionally, eGFRcys shared another genetic variant (rs6114253) with BIP and MDD (PPH4 > 0.5, Table 3).

**Table 3:** Cross-trait SNPs between 2 mental disorder traits and 5 kidney function biomarker traits that colocalized (PPH4 > 0.5)

Exposure	Outcome	SNP	SNP.PP.H4	Gene
BUN	BIP	rs9290867	0.71958943	LPP-AS2
BUN	MDD	rs9290867	0.73130115	LPP-AS2
eGFRcys	BIP	rs6114253	0.76291143	CST1
eGFRcys	MDD	rs6114253	0.66571008	CST1

Furthermore, we found an overlap of SNP rs6114253 between the colocalization and MTAG results. Although rs6114253 did not meet our selection threshold (single trait P < 5 × 10^–4^), the MTAG analysis indicated that rs6114253 represented a significant signal (P_mtag_ = 4.05 × 10^–296^) associated with eGFRcys (P = 6.03 × 10^–277^) in the context of BIP (P = 7.23 × 10^–1^) and MDD (P = 5.94 × 10^–1^).

### TWAS and Tissue‐specific expression analysis

To explore the potential shared gene-tissue associations between kidney function biomarker traits and mental disorders, we also conducted a TWAS analysis using GTEx V8 gene expression data. Our analysis revealed 2476 shared tissue–gene pairs (FDR < 0.05) between 5 kidney function biomarker traits and 2 mental disorders, most of which were observed in the nervous, cardiovascular, exo-/endocrine system and digestive systems (Additional files 4, 5, 6: Supplementary Table 4–6). Notably, we observed 249 significant tissue–gene pairs for BIP and BUN, 489 pairs for MDD and BUN, 536 pairs for BIP and eGFRcrea, 396 pairs for MDD and eGFRcrea, 357 pairs for BIP and eGFRcys, 448 pairs for MDD and eGFRcys (Additional files 6, 7, 8: Supplementary Table 6–8). Among these pairs, several notable associations were found. For BIP and kidney function biomarker traits, the strongest expression–trait association was observed at *SCGB1B2P* (the strongest association at the heart left ventricle, FDR_TWAS_ = 3.19 × 10^–9^), which is a member of secretoglobin family [48]. In addition, RPL13 (the strongest association at stomach, FDR_TWAS_ = 2.23 × 10^–7^) and *LINC00189* (the strongest association at the hypothalamus, FDR_TWAS_ = 1.46 × 10^–6^) were also TWAS significant. For MDD and kidney function biomarker traits, the strongest expression–trait association was observed at *ECHDC1* (the strongest association at the cells cultured fibroblasts, FDR_TWAS_ = 3.19 × 10^–9^), which encodes ethylmalonyl-CoA decarboxylase 1 [49]. Among these 2476 shared tissue–gene pairs, 3 novel genes: *SUFU*, *IBSP*, and *PTPRJ*, were also identified in MTAG.

To gain further insights into the enriched expression of shared genes between the 5 kidney function biomarker traits and the 2 mental disorder traits, we further conducted a tissue-specific enrichment analysis. A total of 5 tissues were found with an enriched expression of shared genes between 5 kidney function biomarker traits and 2 mental disorder traits. The mainly enriched tissues contained the kidney cortex, kidney medulla, liver, pancreas, and skeletal muscle. We found that common pathways in KEGG and GO for genes shared between 5 kidney function biomarker traits and 2 mental disorder traits included ribonucleotide binding, biosynthetic process, gene expression, mitochondrion, cell differentiation and immune systems diseases (Additional files 7, 8, 9, 10: Supplementary Table 7–10, Additional file 12: Supplementary Fig. 11–20).

### Mendelian randomization results

Under the primary genome-wide significance *P*-value threshold of P < 5 × 10^–5^, a total of 447, 340, 300, 680, 374, 74, 258 instrumental SNPs were retained for BIP, MDD, BUN, eGFRcrea, eGFRcys, UACR, and urate, respectively (Additional file 11: Supplementary Table 11).

**Fig. 2:**
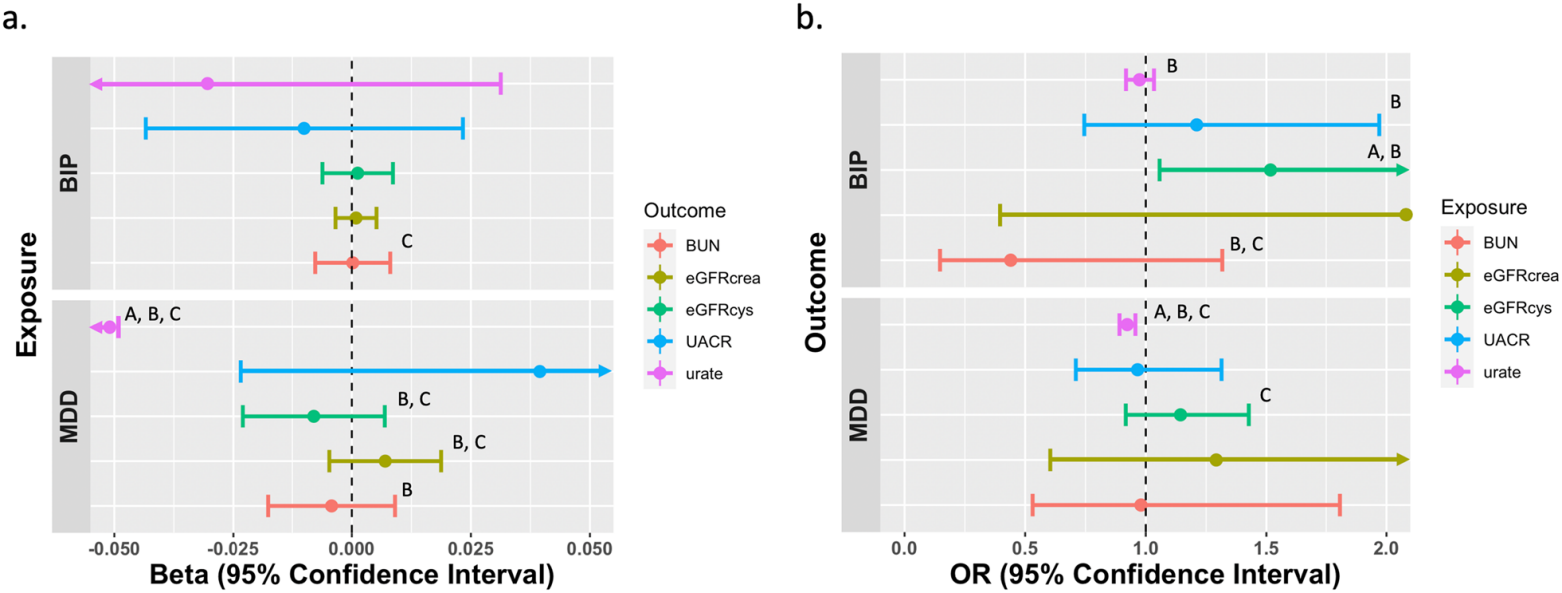
Bidirectional MR results using mode-based estimate (MBE) method. **a** Forest plot of the main MR study investigating the causal effect of 2 mental disorders on 5 kidney function biomarker traits using the MBE method. **b** Forest plot of the main MR study investigating the causal effect of 5 kidney function biomarker traits on 2 mental disorders using the MBE method. A *P*-value of MBE < 0.05, B *P*-value of ConMix < 0.05, C *P*-value of MR-Egger < 0.05. BIP: bipolar disorder; MDD: major depressive disorder; BUN: blood urea nitrogen; eGFRcys: cystatin C-based estimated glomerular filtration rate; UACR: urine albumin-creatinine ratio; eGFRcrea: creatinine-based estimated glomerular filtration rate

The bidirectional Mendelian randomization revealed causal relationships of eGFRcys (OR_MBE_ = 1.517, 95% CI: 1.057 – 2.178, P = 2.40 × 10^–2^) and UACR on BIP, as well as MDD on eGFRcys, which are consistent with the previous LDSC results. Additionally, Bidirectional MR analysis also revealed causal relationships of urate on BIP, as well as MDD on eGFRcrea. Furthermore, we also found a bidirectional causal relationship between MDD and urate (forward Beta_MBE_ = – 0.538, 95% CI: -0.795 – (-0.282), P = 3.98 × 10^–5^; reverse OR_MBE_ = 0.923, 95% CI: 0.891 – 0.957, P = 1.00 × 10^–3^). However, the MR results showed no significant causal relationship of BIP on eGFRcrea, eGFRcys, UACR, and urate (all P > 0.05). Also no siginificant assosiation of BUN, eGFRcrea, and UACR on MDD (all P > 0.05, Fig. 2).

## Discussion

To our knowledge, this is the first study to identify genome-wide genetic correlation and shared genetic variants of kidney function biomarker traits on BIP and MDD. Our findings reveal significant causal relationships between these traits. Cross-trait meta-analysis identified 5 to 19 independent significant loci that were found shared among 10 pairs of 5 kidney function biomarkers traits and 2 mental disorders. we would highlight 4 overlapped loci across 10 pairs of all the traits, *including GCKR*, *MUC1*, *HSPA1A* and *KRT18P1*. Furthermore, we found 3 novel genes identified by both MTAG and TWAS, including *PTPRJ*, *IBSP* and *SUFU*. TWAS analysis showed that the shared genes between kidney function biomarkers and mental disorders might express via the nervous, cardiovascular, exo-/endocrine system and digestive systems. Pathway analysis showed the immune system could play a role between function biomarkers and mental disorders.

Impaired kidney function biomarkers such as declined BUN and eGFRcrea, have been identified as independent risk factors for mental disorders. In LDSC analysis, we found significant genetic correlations of BIP with eGFRcrea, eGFRcys, and UACR, as well as between MDD and eGFRcys. The further conducted bidirectional Mendelian randomization confirmed causal relationships of eGFRcys and UACR on BIP, as well as MDD on eGFRcys, supporting the findings from LDSC analysis. Moreover, bidirectional MR analysis also revealed causal relationships of BUN and urate on BIP, which align with the previous epidemiological evidence [50]. For serum urate, the results suggested that the lower urate had a genetic causal effect on a higher risk of BIP at the population level, which supported previous observational studies [51]. Consequently, our results underscore the importance of diligent monitoring for patients with decreased urate levels, not only for other chronic and metabolic complications but also for indications of BIP. This necessitates comprehensive assessments, including physical examinations and psychological evaluations, to facilitate early detection and intervention. Furthermore, we also found a bidirectional causal relationship between MDD and urate, which is consistent with previous studies. For example, a previous clinical cross-sectional study suggested that low levels of serum urate are associated with a higher prevalence of depression [50], while another genetic correlation study demonstrated that urate could have a deleterious effect on general cognitive function [52].

Among 69 significant loci identified by cross-trait meta-analysis using MTAG, we highlighted 4 overlapped loci among 10 pairs of 5 kidney function biomarkers traits and 2 mental disorders, including the genes *GCKR*, *MUC1*, *HSPA1A* and *KRT18P1*. For *GCKR*, our analysis found it was shared by BIP with BUN, eGFRcrea, eGFRcys and urate, which was agreed by many previous studies. For example, individuals with the T/T genotype of *GCKR* rs1260326 were at a significantly higher risk of CKD [41]. Despite limited evidence linking *GCKR* to BIP, it was observed that the *GCKR* encoding glucokinase regulatory protein which is present in human brains, including in the ventromedial and arcuate nuclei of the hypothalamus, indicating its role in glucose sensing in the central nervous system [53]. *MUC1* was found common to BUN and urate in MTAG, which consistent to the previous evidence of *MUC1* associated with urate, UACR and eGFRcrea [44, 45, 54, 55]. MUC1 was also found related to central nervous system diseases such as multiple sclerosis [56]. *HSPA1A* was found to be associated with a poorer response to antidepressants in MDD (*P* = 0.005) [57]. Our study found *HSPA1A* was common to eGFRcrea, eGFRcys and MDD in MTAG, indicating a potential mechanism through which renal dysfunction affects MDD via *HSPA1A*. *KRT18P1* was also associated with MDD and neuroticism reported by previous GWAS study [58]. The colocalization analysis revealed a shared causal variant (rs9290867) between BUN with MDD and BIP (PPH4 > 0.5). This variant is mapped to *LPP-AS2,* a long non-coding RNA that was recently found associated with glioblastoma. Additionally, the genetic variant (rs6114253) in proximity to *CST1*, which encodes Cystatin SN, was found to be shared between eGFRcys and both BIP and MDD. Further studies are needed to investigate the role of these genes in impaired kidney function and mental disorders.

In the TWAS analysis, we identified 2476 shared tissue–gene pairs between kidney function biomarker traits and 2 mental disorders. Among them, we would like to highlight 3 novel genes which were significant both in MTAG and TWAS results, including *PTPRJ* (P_mtag_ = 2.91 × 10^–8^, P_TWAS_BIP_ = 2.08 × 10^–3^, P_TWAS_eGFRcrea_ = 5.16 × 10^–4^), *IBSP* (P_mtag_ = 4.41 × 10^–11^, P_TWAS_BIP_ = 3.44 × 10^–2^, P_TWAS_BUN_ = 5.85 × 10^–4^) and *SUFU* (P_mtag_ = 1.57 × 10^–12^, P_TWAS_MDD_ = 3.49 × 10^–2^, P_TWAS_eGFRcys_ = 1.57 × 10^–6^). *PTPRJ* and *IBSP* were both found to be share between BIP and kidney function biomarker traits (eGFRcrea, BUN) in brain cortex, *PTPRJ* encodes a receptor-type protein tyrosine phosphatase[60]. Studies have shown that *PTPRJ* was associated with N-Acetyl-aspartyl-glutamate, which could regulate peptide neurotransmitters in the mammalian nervous system [61, 62]. Therefore, *PTPRJ* could be a potential target for mental disorders. *IBSP* is a member of the small integrin-binding ligand N-linked glycoprotein family [63, 64]. Few studies have reported that *IBSP* was found to be associated with the nervous system and kidney function. Our study suggested that *IBSP* could be a potential site for regulating renal function and mental disorders. *SUFU* was shared between MDD and eGFRcys in brain basal ganglia, which is a negative regulator of hedgehog signaling. The mutation of *SUFU* predisposes to the sonic hedgehog medulloblastoma [65]. GO biological process shows that *SUFU* plays an important role in the regulation of cell differentiation which corroborates the reports of previous studies [65–67]. *SUFU* was also reported to be altered in low-grade or high-grade meningioma or were in the same pathways as known meningioma drivers [68].

Post-GWAS functional analyses provided biological insights into the shared genes between kidney function and mental disorders traits. GTEx tissue enrichment analysis identified shared genes that were significantly enriched in several tissues, including the kidney cortex, kidney medulla, liver, pancreas, and skeletal muscle. KEGG pathway analysis showed that a set of 115 genes is enriched in immunity‐related signaling functions, consistent with the immune system being the major driver of mental disorders [69–73]. Epidemiologic and genetic studies have suggested the existence of a kidney-brain axis, where kidney damage causally influences the brain cortical structure [28, 69, 74]. This comprehensive evaluation of genetic correlation and causality between mental disorders and kidney function provides new insights into the shared loci and biological mechanisms underlying this comorbidity. The importance of impaired kidney function as a predictor for the risk of mental disorders is increasingly being recognized [75].

This study has several notable strengths. Firstly, it is the first analysis to identify the shared genetic architecture of kidney function and mental disorders using a large-scale observational GWAS dataset (sample size up to 1,004,040). Secondly, by employing multiple test correction such as FDR, we minimized the influence of confounding factors, which may improve the reliability of our findings. Lastly, we identified 3 novel independent loci (*PTPRJ*, *IBDP*, and *SUFU*) that are associated with both kidney function and mental disorders using multi-comics statistical methods such as MTAG and TWAS, which may contribute to a better understanding of the underlying mechanisms.

We also acknowledge several potential limitations in this study. Firstly, the 5 kidney function biomarker traits may not capture the full spectrum of the impairment of kidney function. Nevertheless, we attempted to capture renal function from multiple perspectives by using various blood and urine indicators. Secondly, horizontal pleiotropy may exist between exposure and outcome, which could reduce the statistical power of traditional MR analysis. Consequently, we employed robust MR methods such as MBE and ConMix, to strengthen the validity of our findings. Thirdly, the study population in this research was predominantly of European ancestry. Therefore, the results of this study should be interpreted with caution when considering other ancestral populations.

## Conclusions

Understanding the genetic overlap between kidney function biomarkers and mental disorders may be beneficial to the management of both conditions. Our study provides evidence of significant genetic correlations and causal relationships between kidney function biomarkers and mental disorders. Shared genetic variants were mapped to improve resolution and identify potential shared causal variants with exonic missense polymorphisms. We also found multiple potential common biological mechanisms, which can advance our understanding of the connection between kidney function biomarkers and mental disorders. Such shared genes and pathways might serve as common drug targets in impaired kidney function, BIP and MDD.

### Supplementary Information

**Additional file 1**. **Supplementary Table 1.** Summary of GWAS data.

**Additional file 2**. **Supplementary Table 10.** Shared gene set between 2 mental disorder traits and 5 kidney function biomarker traits in KEGG.

**Additional file 3**. **Supplementary Table 11.** Plink clumping using R2<0.1, Bidirectional Mendelian randomization results for the causal association between 2 mental disorder traits and 5 kidney function biomarker traits.

**Additional file 4**. **Supplementary Table 2.** Genetic correlation between 2 mental disorder traits and 5 kidney function biomarker traits.

**Additional file 5**. **Supplementary Table 3.** Frequency of overlaped gene annotation of MTAG results.

**Additional file 6**. **Supplementary Table 4.** Significant overlap transcriptome-wide association analysis results between2 mental disorder traits and 5 kidney function biomarker traits (FDR<0.05).

**Additional file 7**. **Supplementary Table 5.** Transcriptal-wide association analysis forBIP and MDD using GTEx tissue expression (FDR<0.05).

**Additional file 8**. **Supplementary Table 6.** Transcriptal-wide association analysis for kidney function biomarker traits using GTEx tissue expression (false discovery rate (FDR)<0.05).

**Additional file 9**. **Supplementary Table 7.** Biological process of the shared gene set between 2 mental disorder traits  and 5 kidney function biomarker traits in Gene Ontology (GO) terms

**Additional file 10**. **Supplementary Table 8.** Cellular component  of the shared gene set between 2 mental disorder traits  and 5 kidney function biomarker traits in Gene Ontology (GO) terms.

**Additional file 11**. **Supplementary Table 9.** Molecular function of the shared gene set between 2 mental disorder traits  and 5 kidney function biomarker traits in Gene Ontology (GO) terms.

**Additional file 12**. Supplementary Figures.

## Data Availability

The datasets used and/or analysed during the current study are available from the corresponding author on reasonable request. All data generated or analysed during this study are included in this published article and its supplementary information files.
