# Supplementary material for: Shared genetic effect of kidney function on bipolar and major depressive disorders: a large-scale genome-wide cross-trait analysis
Source: Hum Genomics. 2024 Jun 11;18:60. doi: 10.1186/s40246-024-00627-3 (PMC11165782; doi:10.1186/s40246-024-00627-3)

Supplementary Figures

**Supplementary Figure 1.** MTAG result between BUN and BIP for independent genome-wide significant association SNPs. The x-axis shows chromosomal position, and the y-axis shows significance on a −log10 scale. The line marks the threshold for genome-wide significance (P = 5E-8).


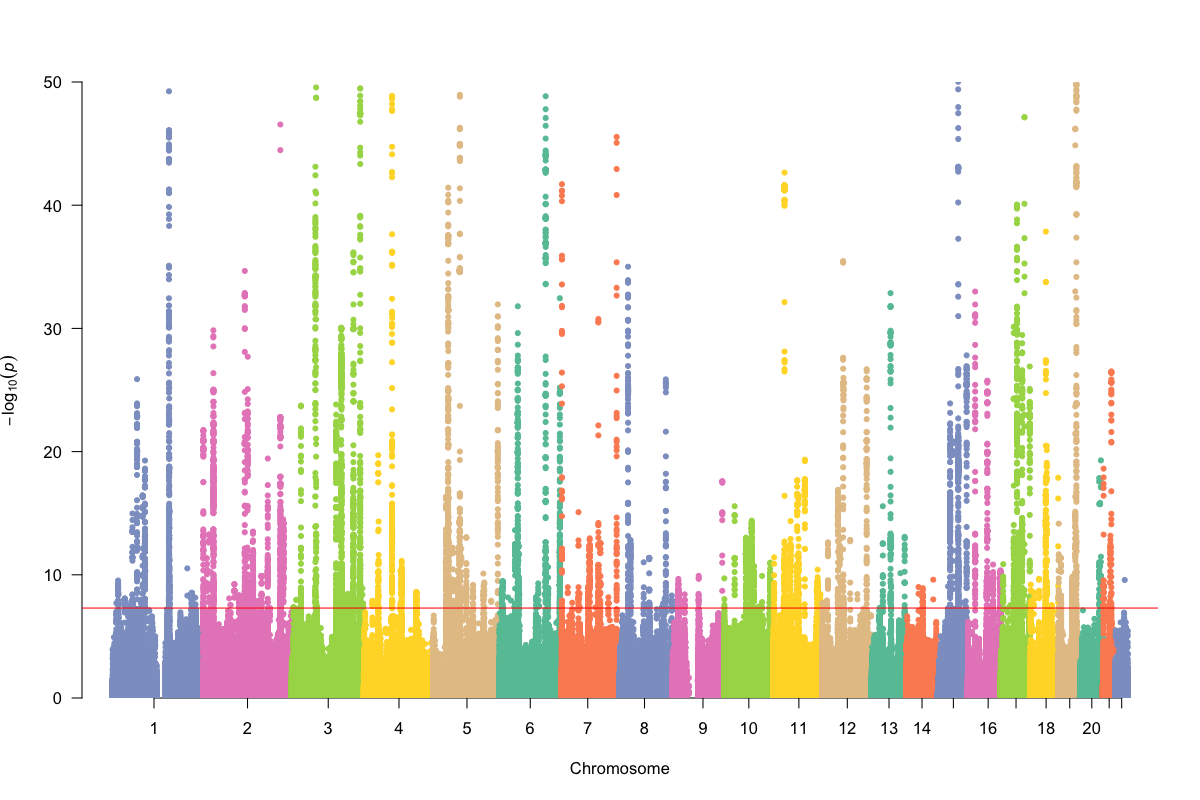


**Supplementary Figure 2.** MTAG result between BUN and MDD for independent genome-wide significant association SNPs. The x-axis shows chromosomal position, and the y-axis shows significance on a −log10 scale. The line marks the threshold for genome-wide significance (P = 5E-8).


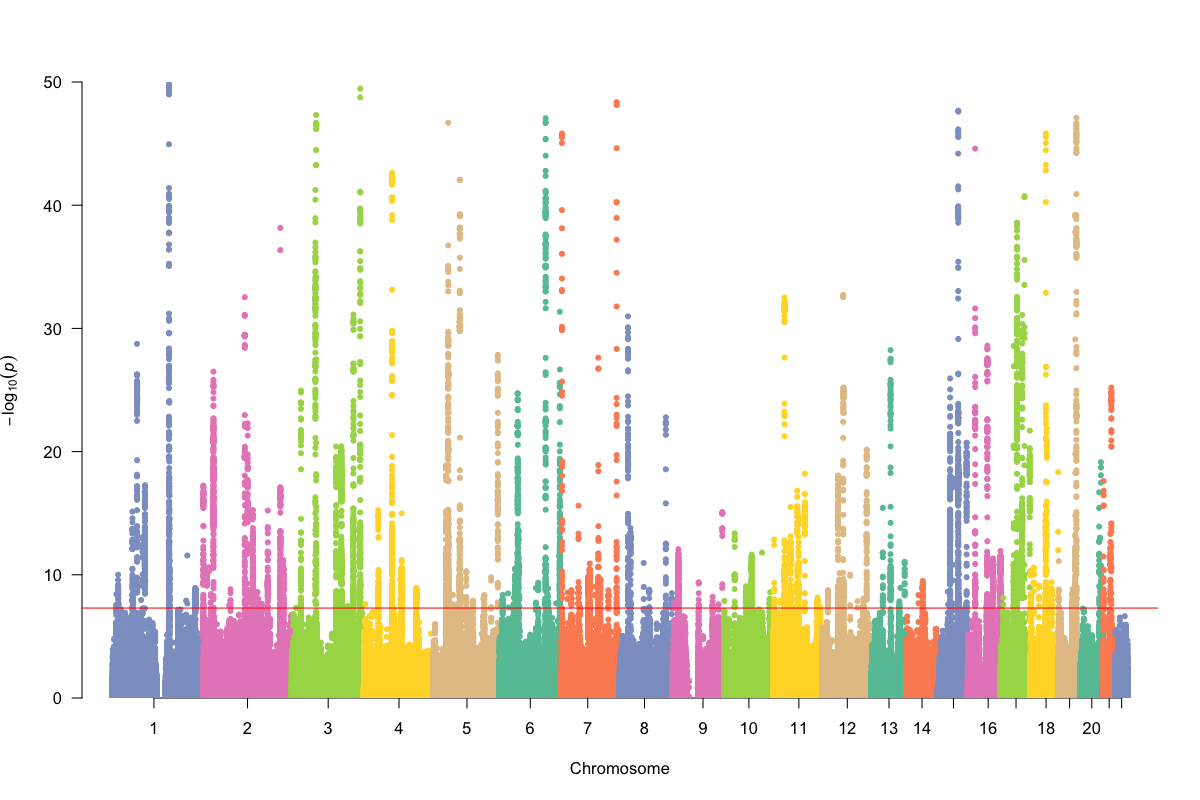


**Supplementary Figure 3.** MTAG result between eGFRcys and BIP for independent genome-wide significant association SNPs. The x-axis shows chromosomal position, and the y-axis shows significance on a −log10 scale. The line marks the threshold for genome-wide significance (P = 5E-8).


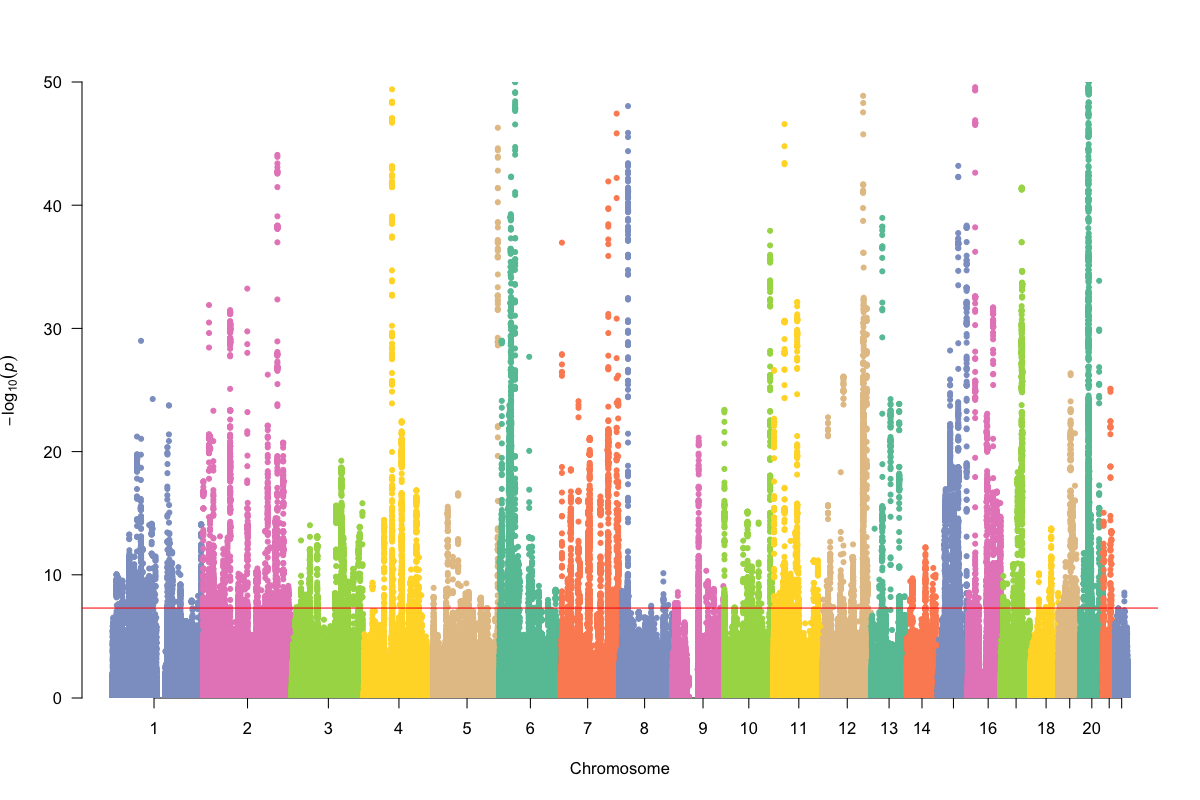


**Supplementary Figure 4.** MTAG result between eGFRcys and MDD for independent genome-wide significant association SNPs. The x-axis shows chromosomal position, and the y-axis shows significance on a −log10 scale. The line marks the threshold for genome-wide significance (P = 5E-8).


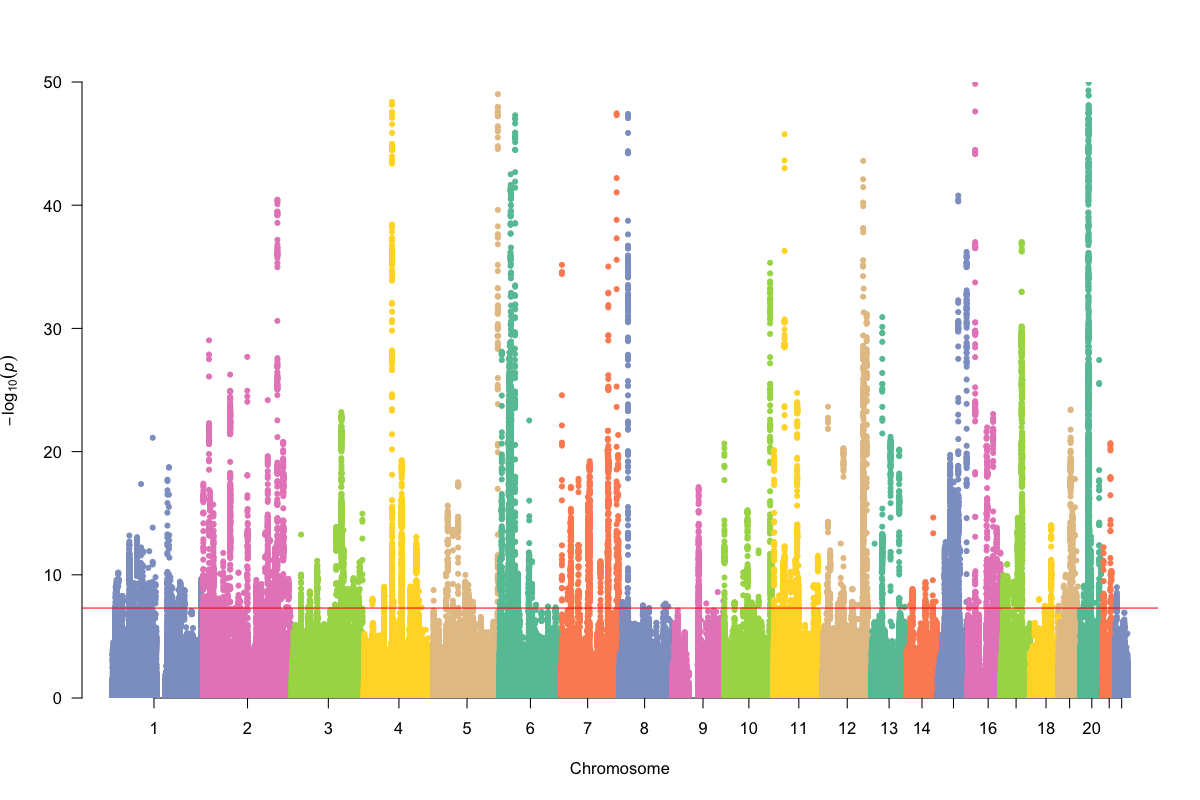


**Supplementary Figure 5.** MTAG result between eGFRcrea and BIP for independent genome-wide significant association SNPs. The x-axis shows chromosomal position, and the y-axis shows significance on a −log10 scale. The line marks the threshold for genome-wide significance (P = 5E-8).


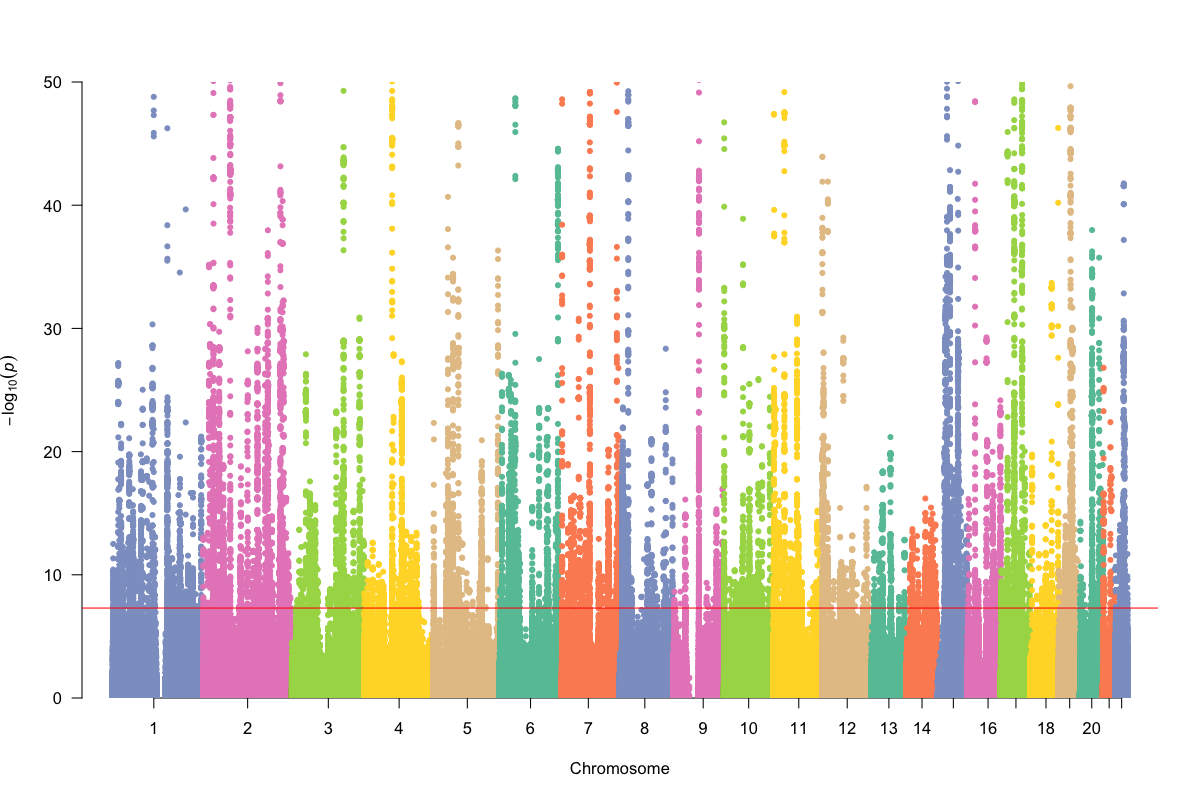


**Supplementary Figure 6.** MTAG result between eGFRcrea and MDD for independent genome-wide significant association SNPs. The x-axis shows chromosomal position, and the y-axis shows significance on a −log10 scale. The line marks the threshold for genome-wide significance (P = 5E-8).


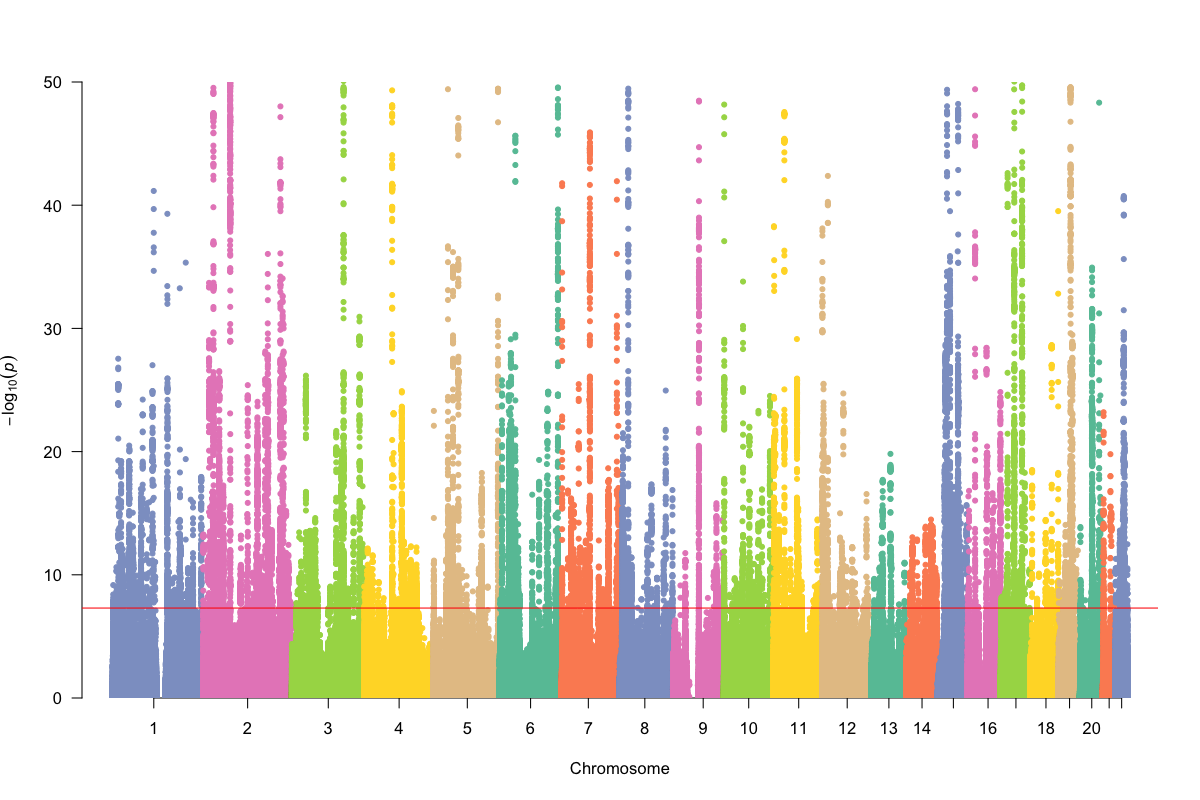


**Supplementary Figure 7.** MTAG result between UACR and BIP for independent genome-wide significant association SNPs. The x-axis shows chromosomal position, and the y-axis shows significance on a −log10 scale. The line marks the threshold for genome-wide significance (P = 5E-8).


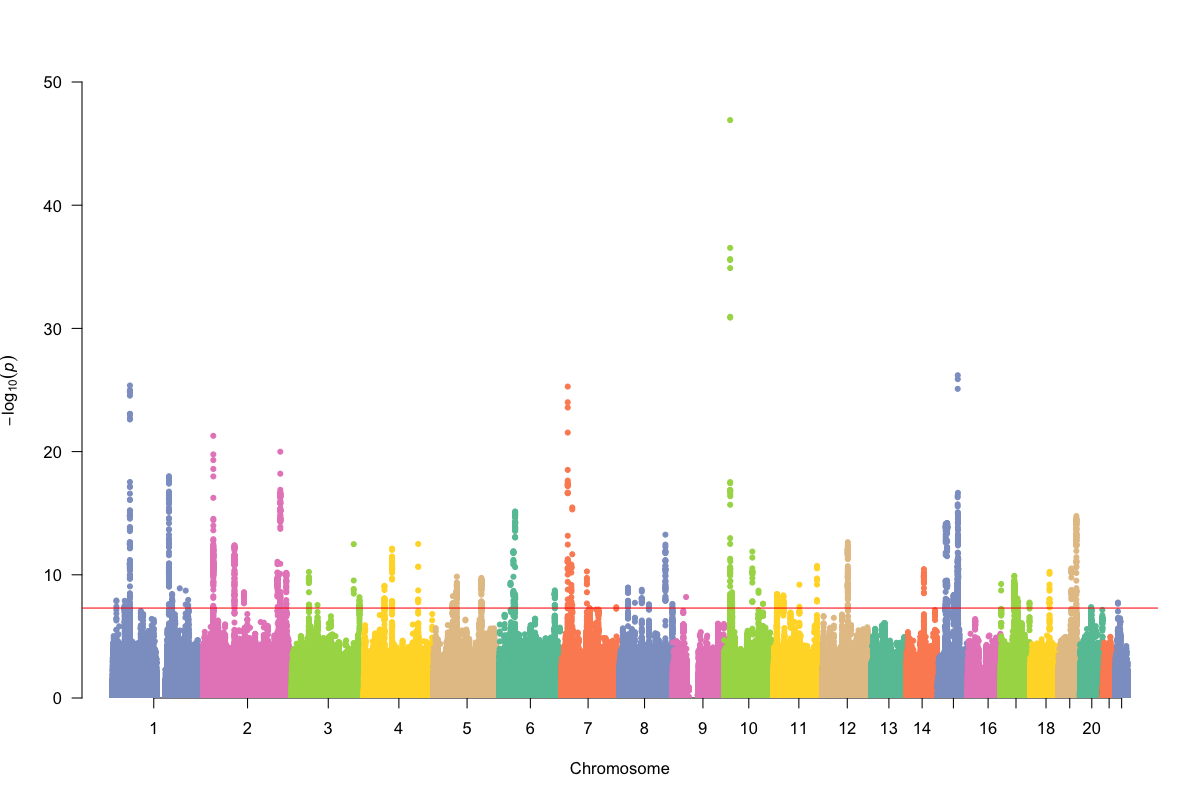


**Supplementary Figure 8.** MTAG result between UACR and MDD for independent genome-wide significant association SNPs. The x-axis shows chromosomal position, and the y-axis shows significance on a −log10 scale. The line marks the threshold for genome-wide significance (P = 5E-8).


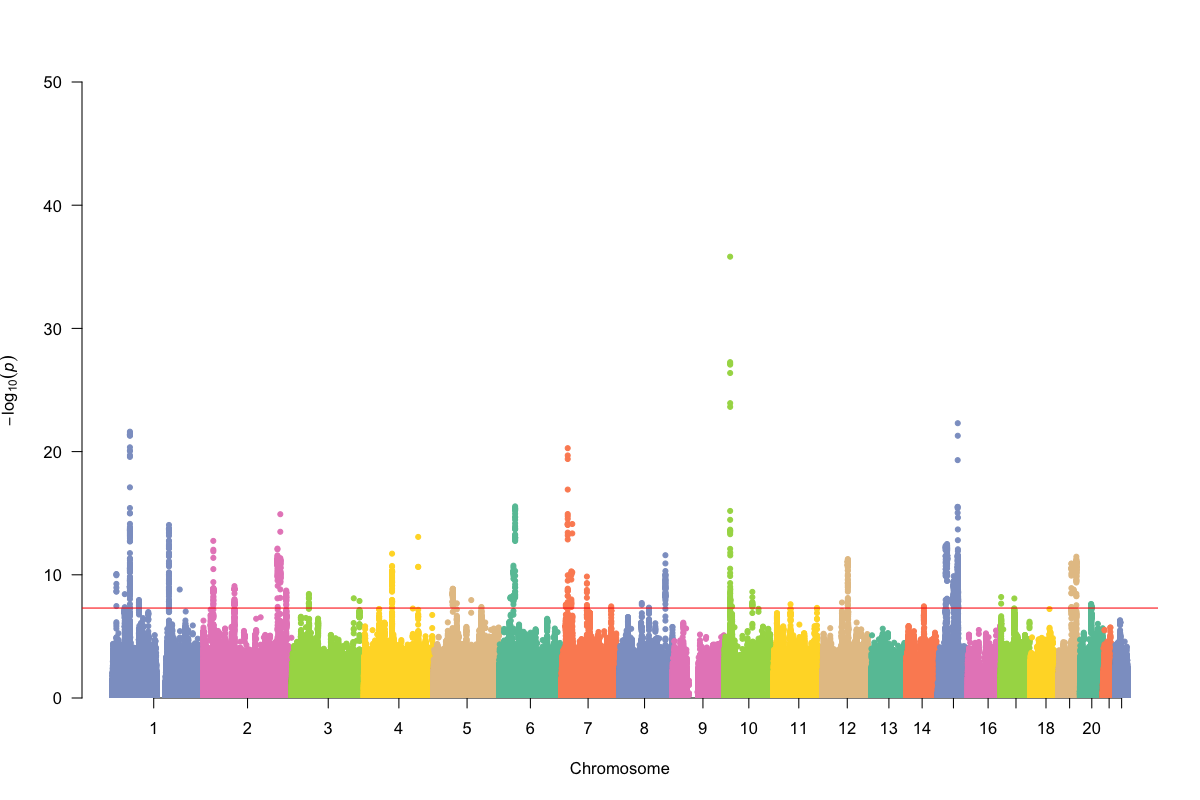


**Supplementary Figure 9.** MTAG result between urate and BIP for independent genome-wide significant association SNPs. The x-axis shows chromosomal position, and the y-axis shows significance on a −log10 scale. The line marks the threshold for genome-wide significance (P = 5E-8).


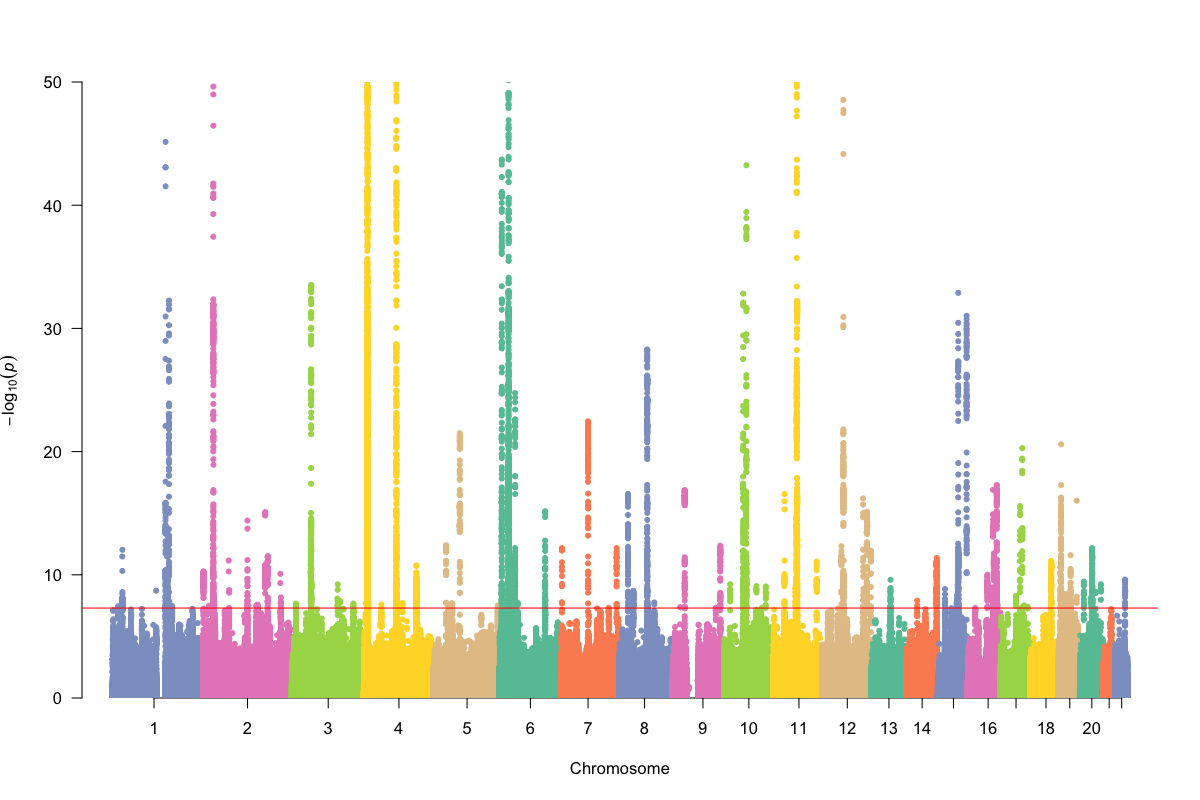


**Supplementary Figure 10.** MTAG result between urate and MDD for independent genome-wide significant association SNPs. The x-axis shows chromosomal position, and the y-axis shows significance on a −log10 scale. The line marks the threshold for genome-wide significance (P = 5E-8).


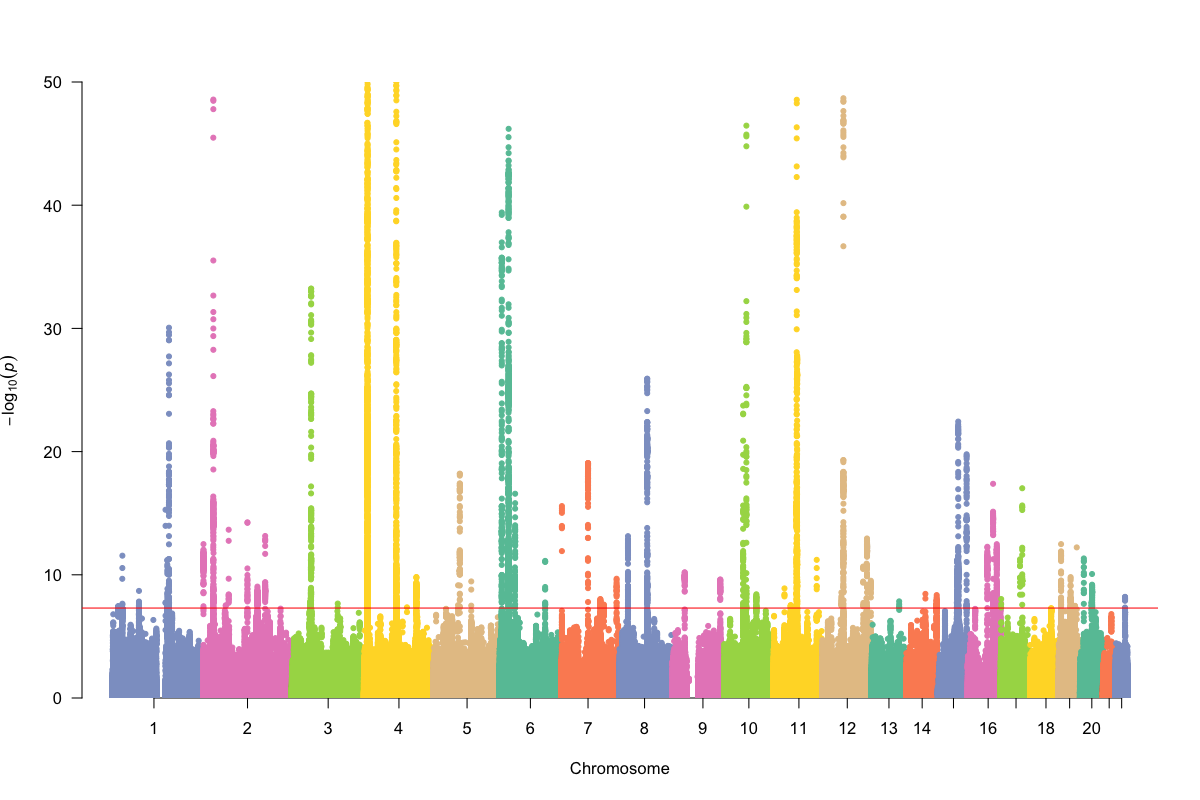


**Supplementary Figure 11.** Gene-based tissue enrichment analysis of the shared genome-wide risk genes between BUN and BIP


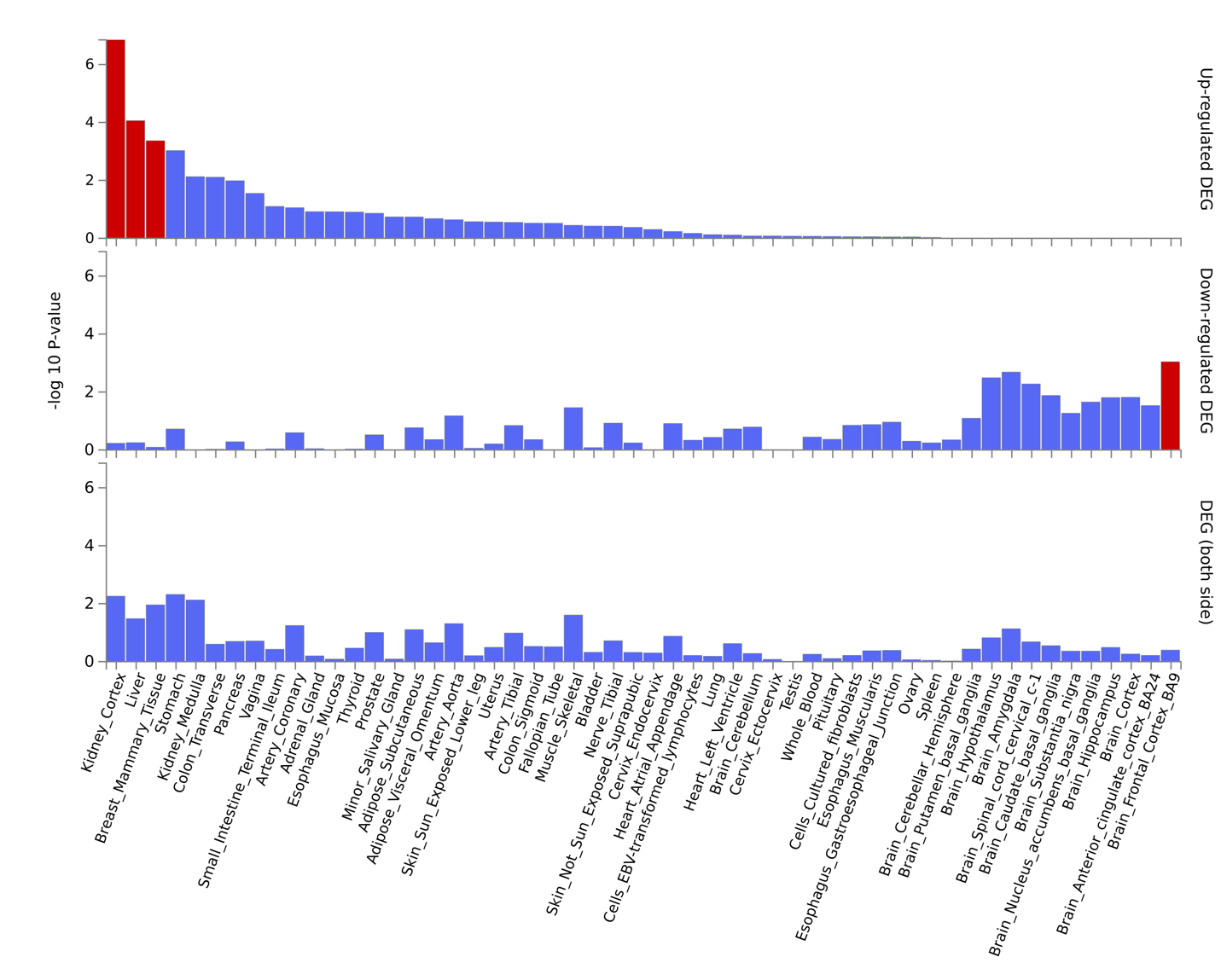


**Supplementary Figure 12.** Gene-based tissue enrichment analysis of the shared genome-wide risk genes between BUN and MDD


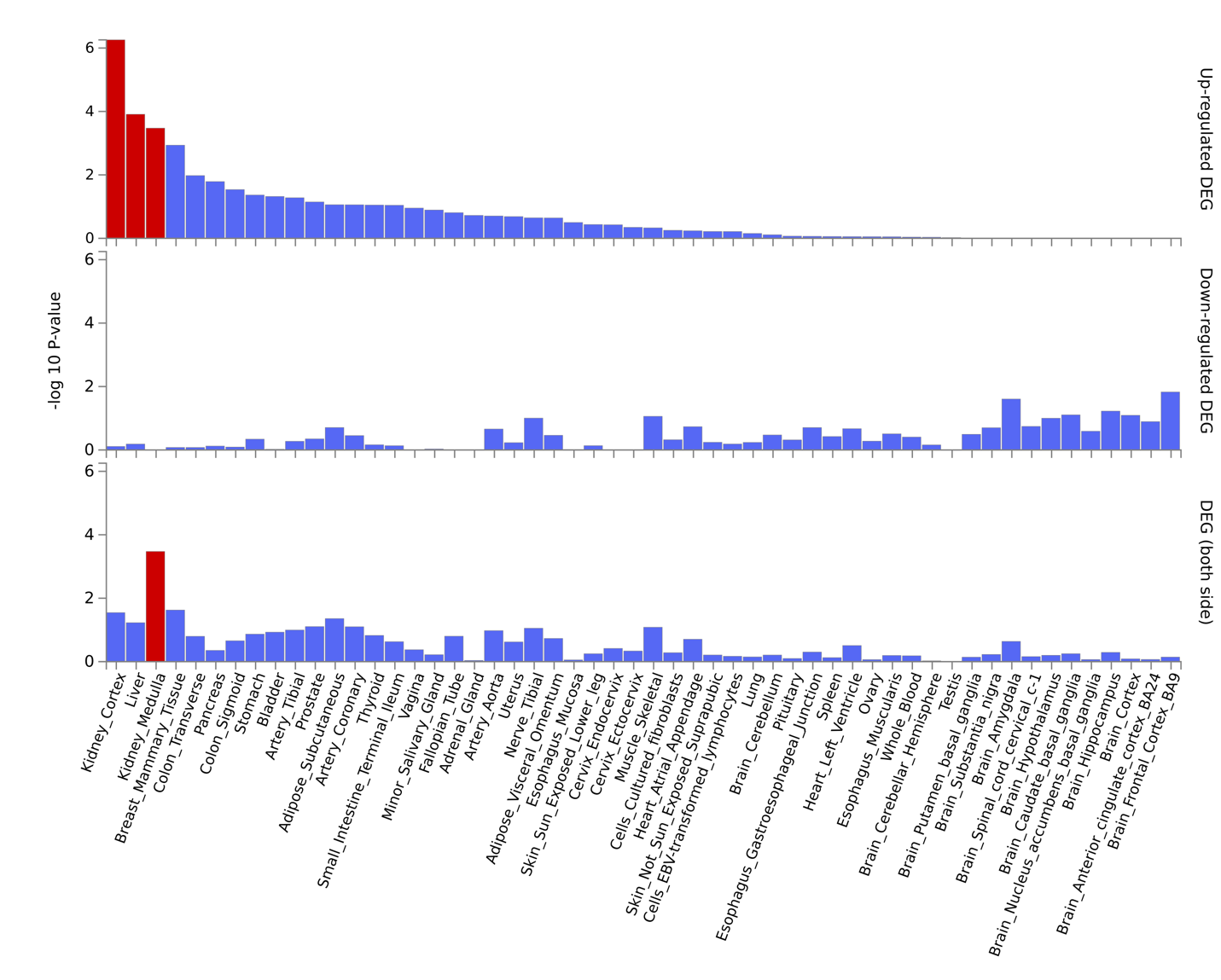


**Supplementary Figure 13.** Gene-based tissue enrichment analysis of the shared genome-wide risk genes between eGFRcys and BIP


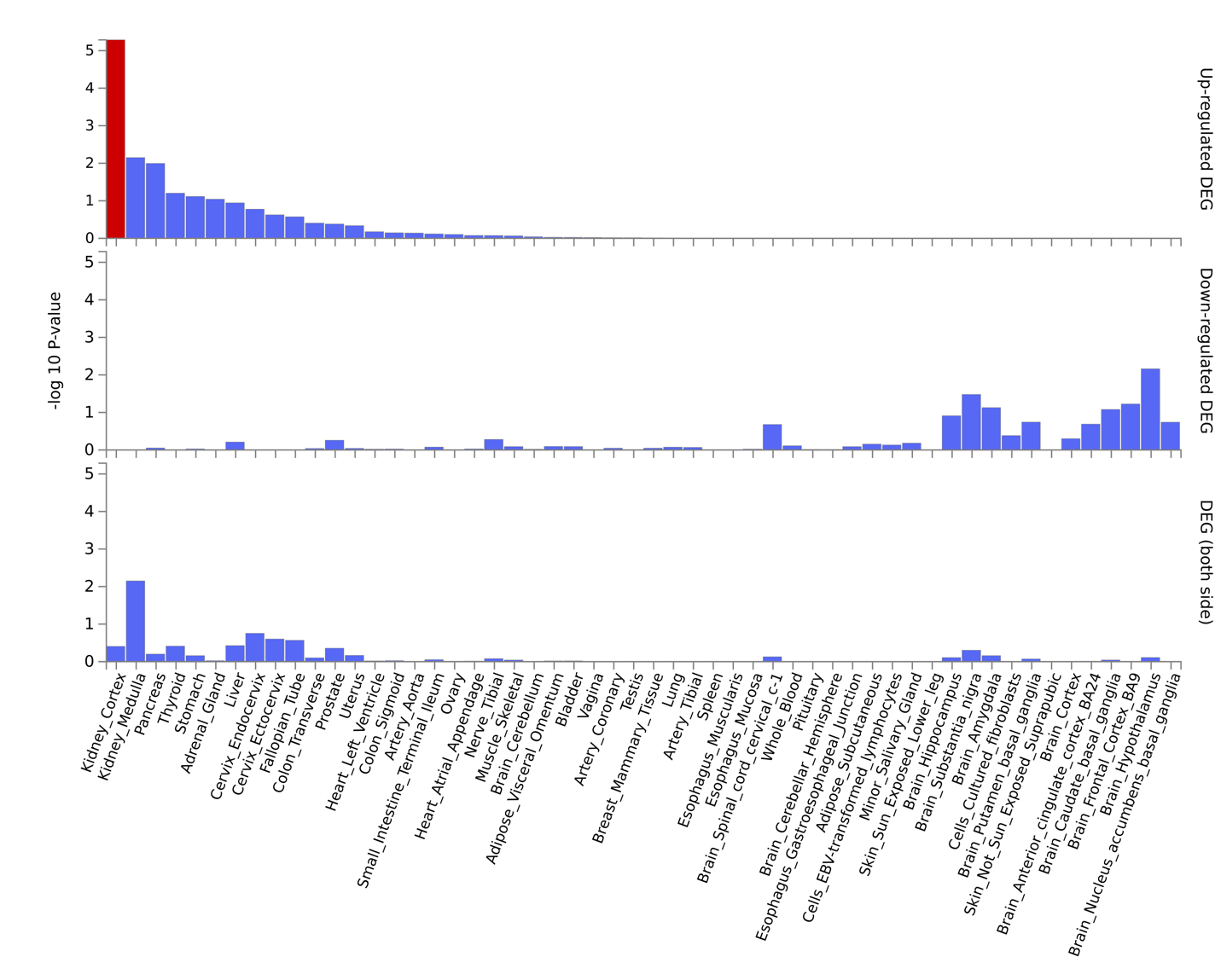


**Supplementary Figure 14.** Gene-based tissue enrichment analysis of the shared genome-wide risk genes between eGFRcrea and BIP


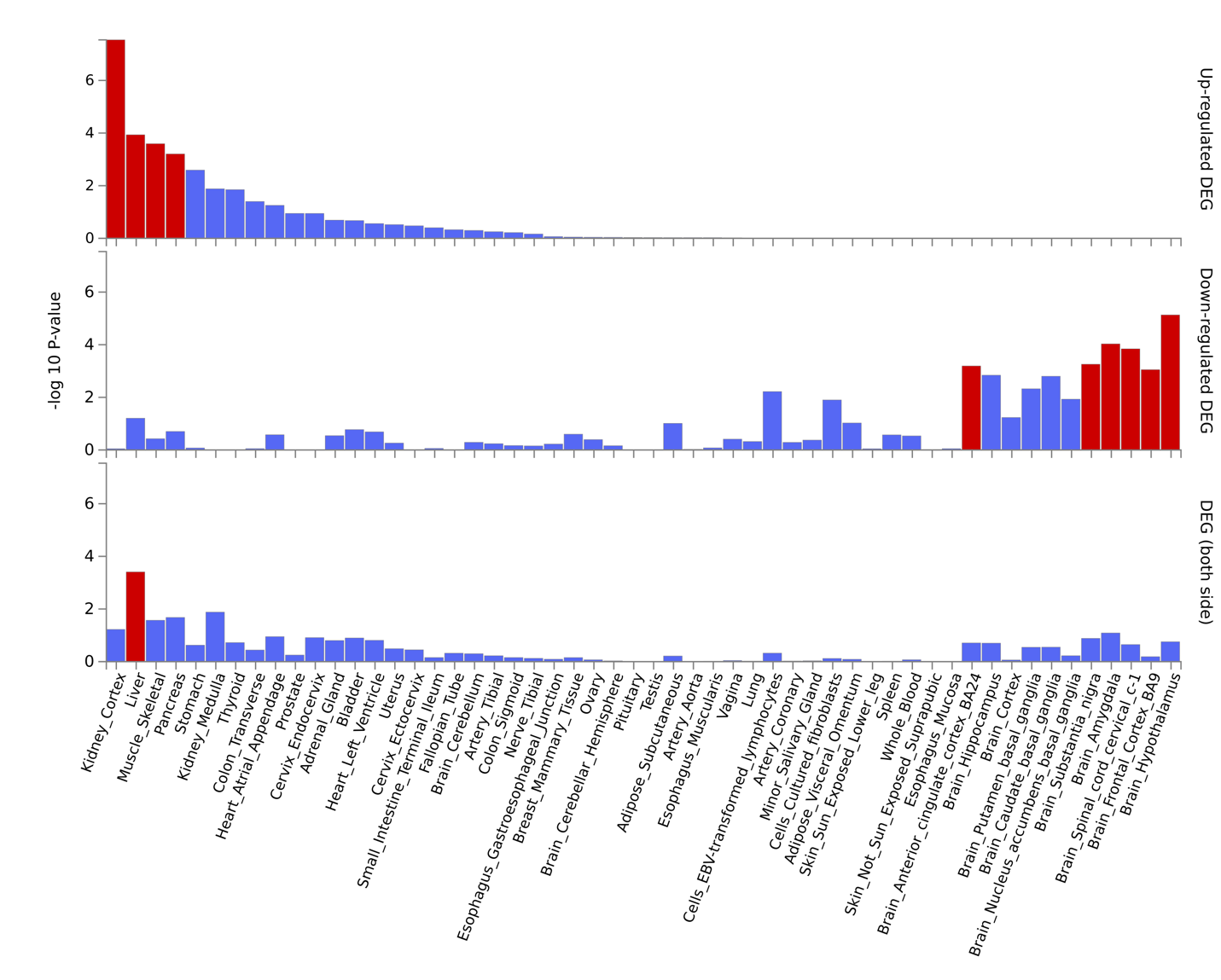


**Supplementary Figure 15.** Gene-based tissue enrichment analysis of the shared genome-wide risk genes between eGFRcrea and BIP


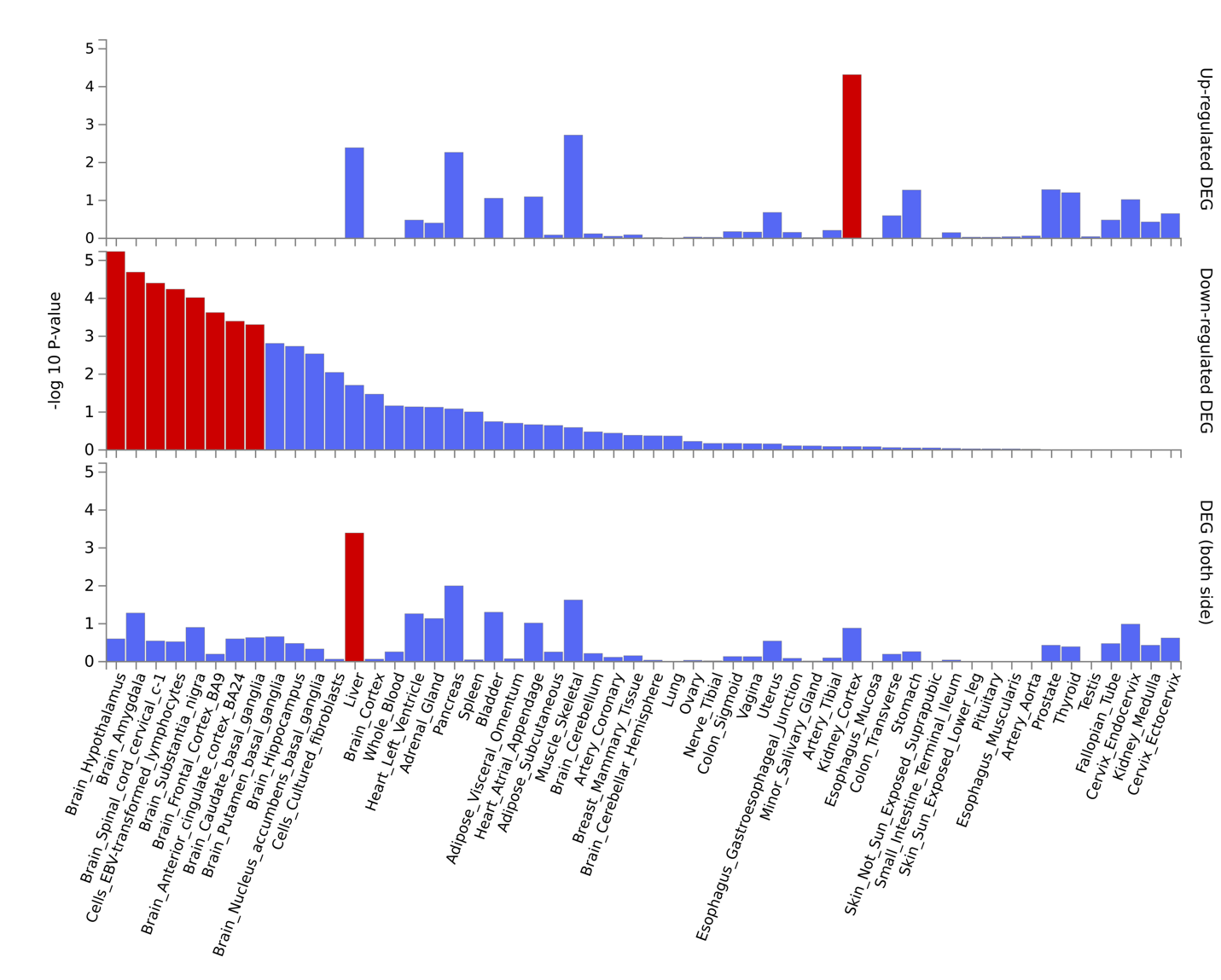


**Supplementary Figure 16.** Gene-based tissue enrichment analysis of the shared genome-wide risk genes between eGFRcys and MDD


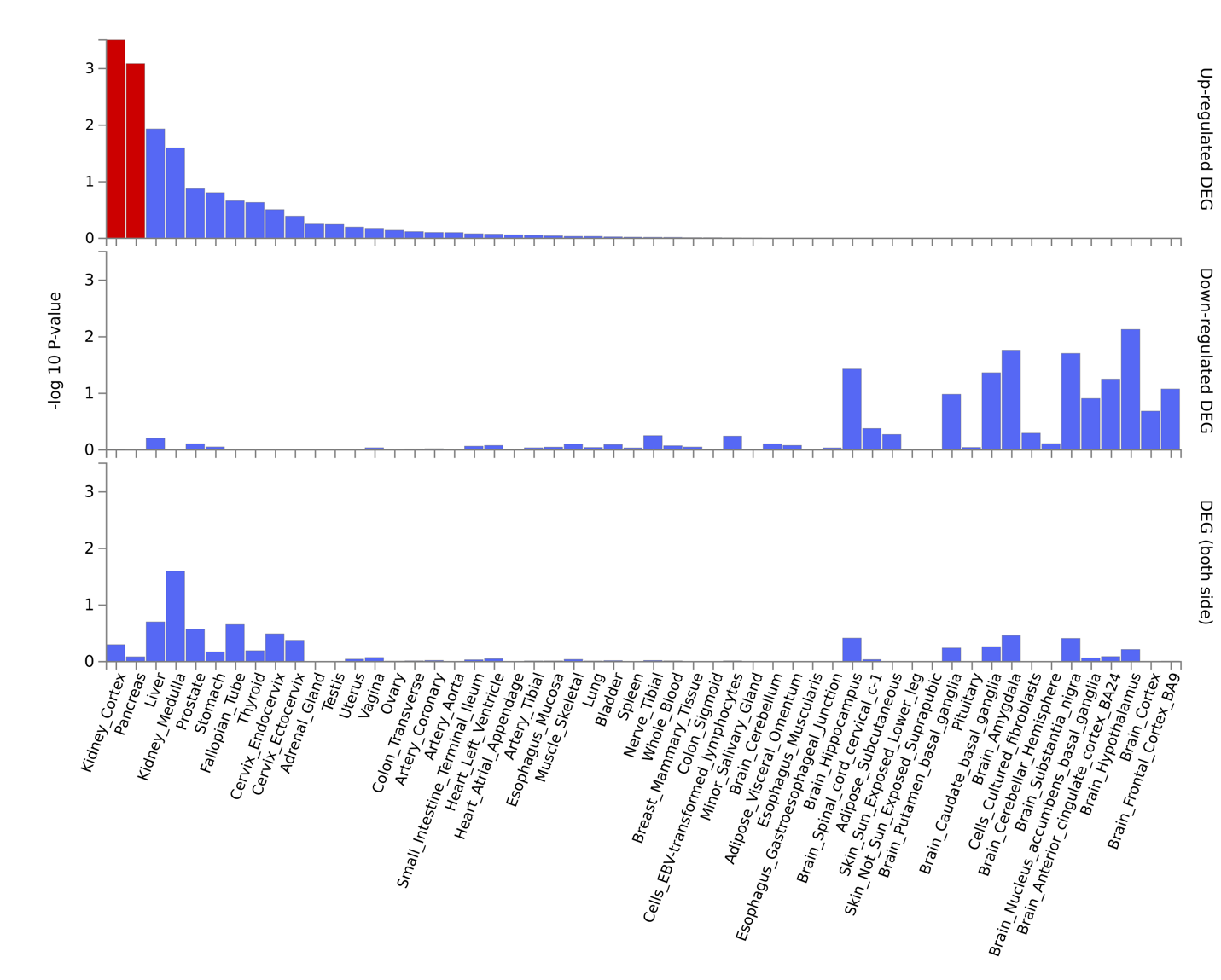


**Supplementary Figure 17.** Gene-based tissue enrichment analysis of the shared genome-wide risk genes between UACR and MDD


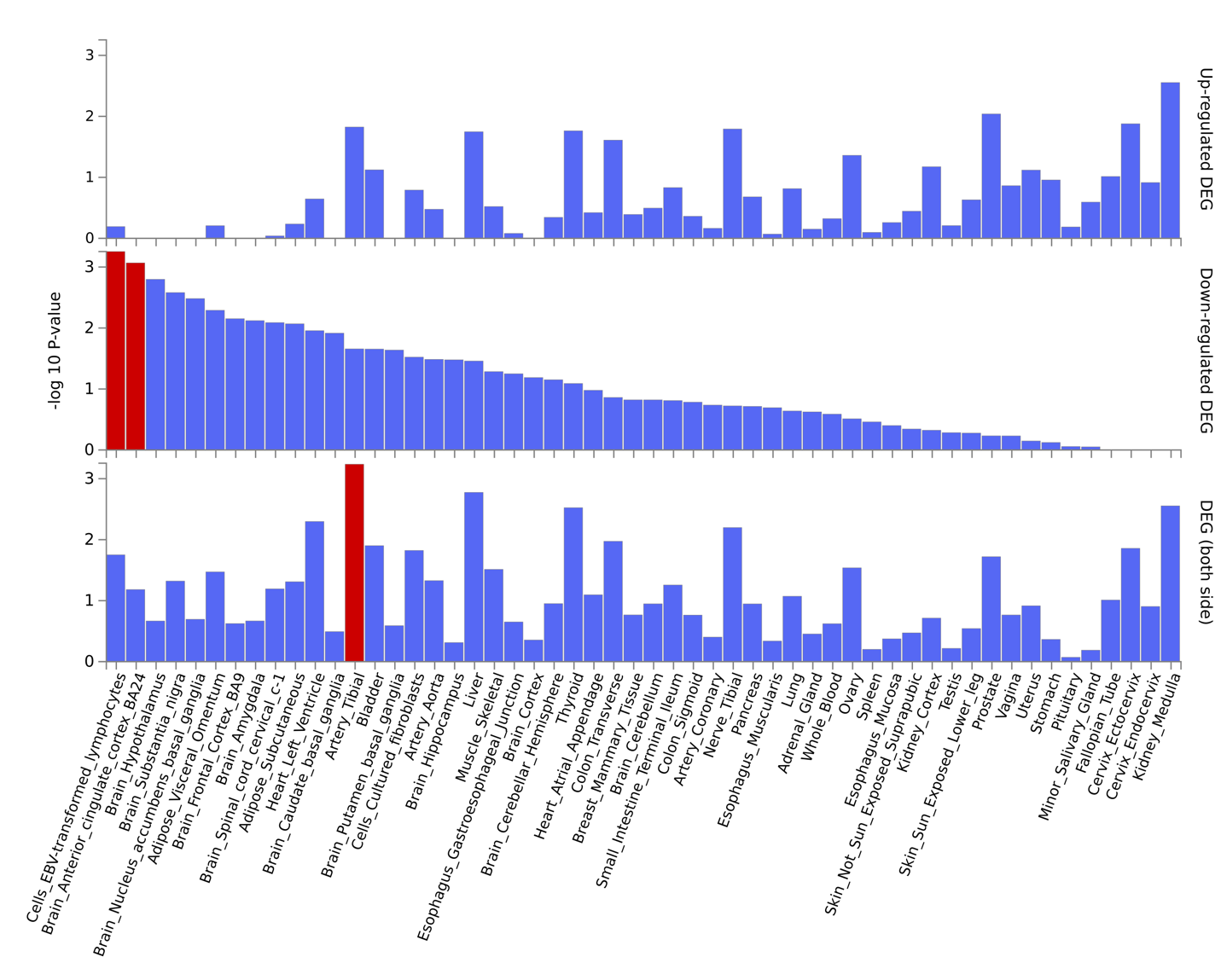


**Supplementary Figure 18.** Gene-based tissue enrichment analysis of the shared genome-wide risk genes between UACR and MDD


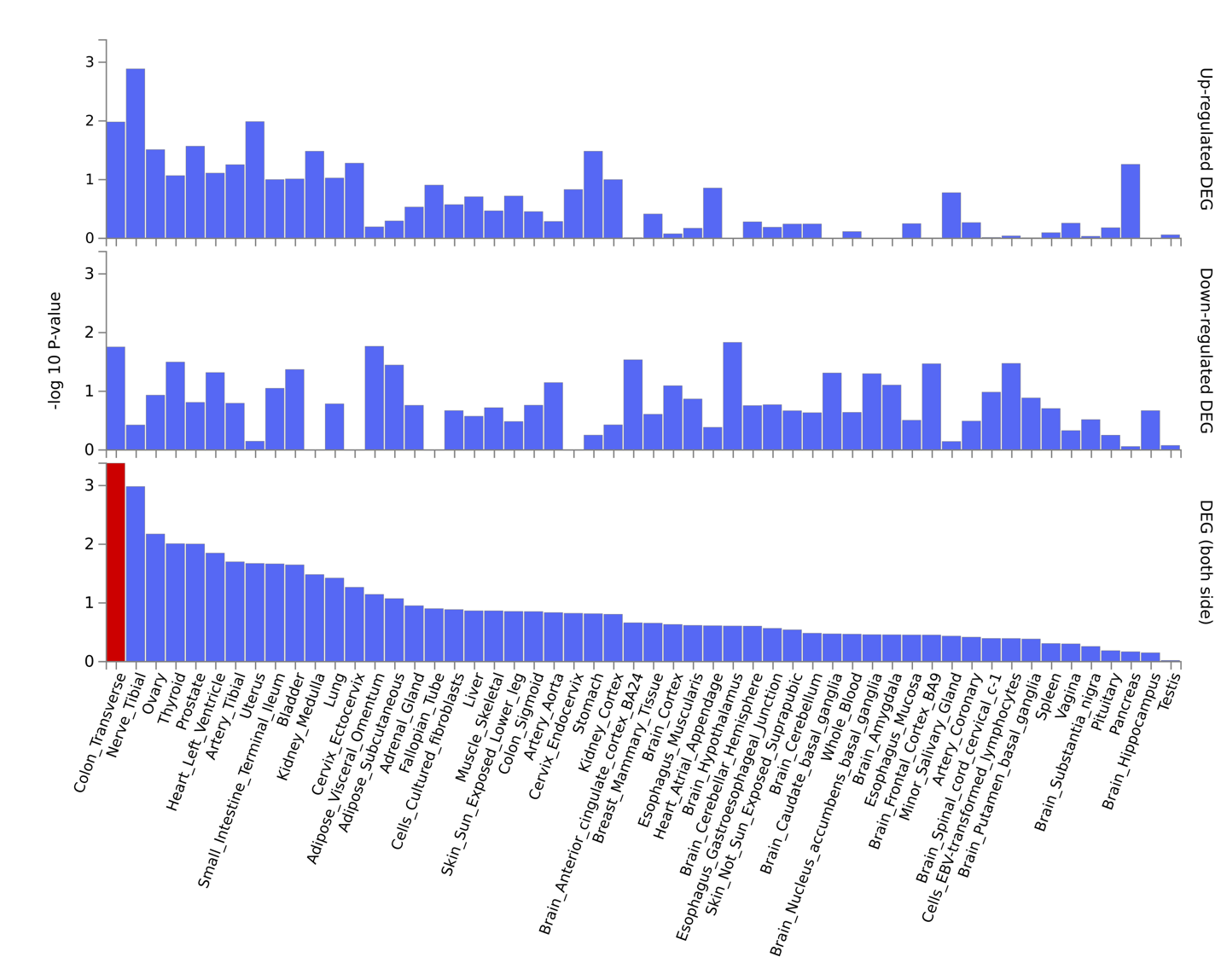


**Supplementary Figure 19.** Gene-based tissue enrichment analysis of the shared genome-wide risk genes between urate and BIP


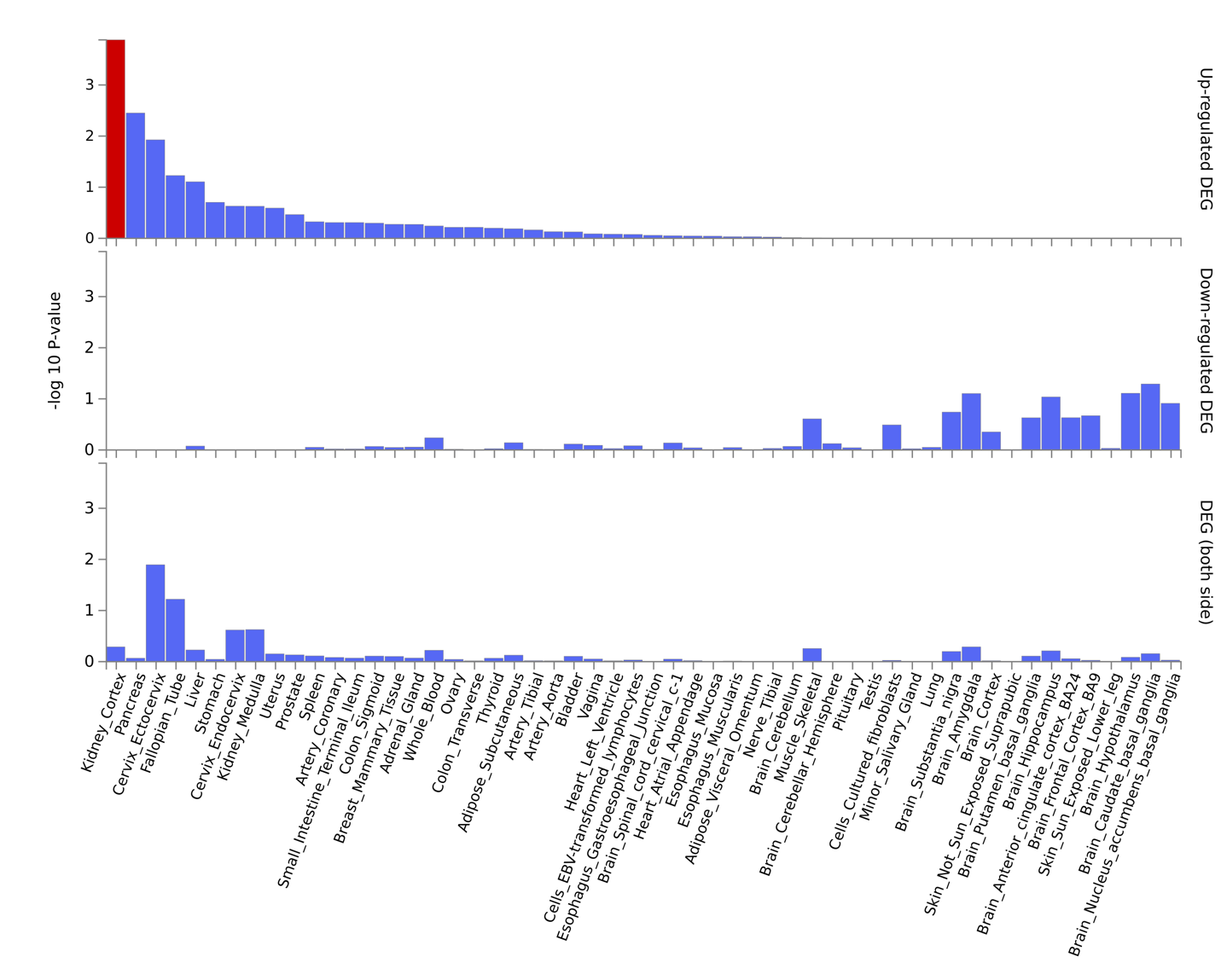


**Supplementary Figure 20.** Gene-based tissue enrichment analysis of the shared genome-wide risk genes between urate and MDD


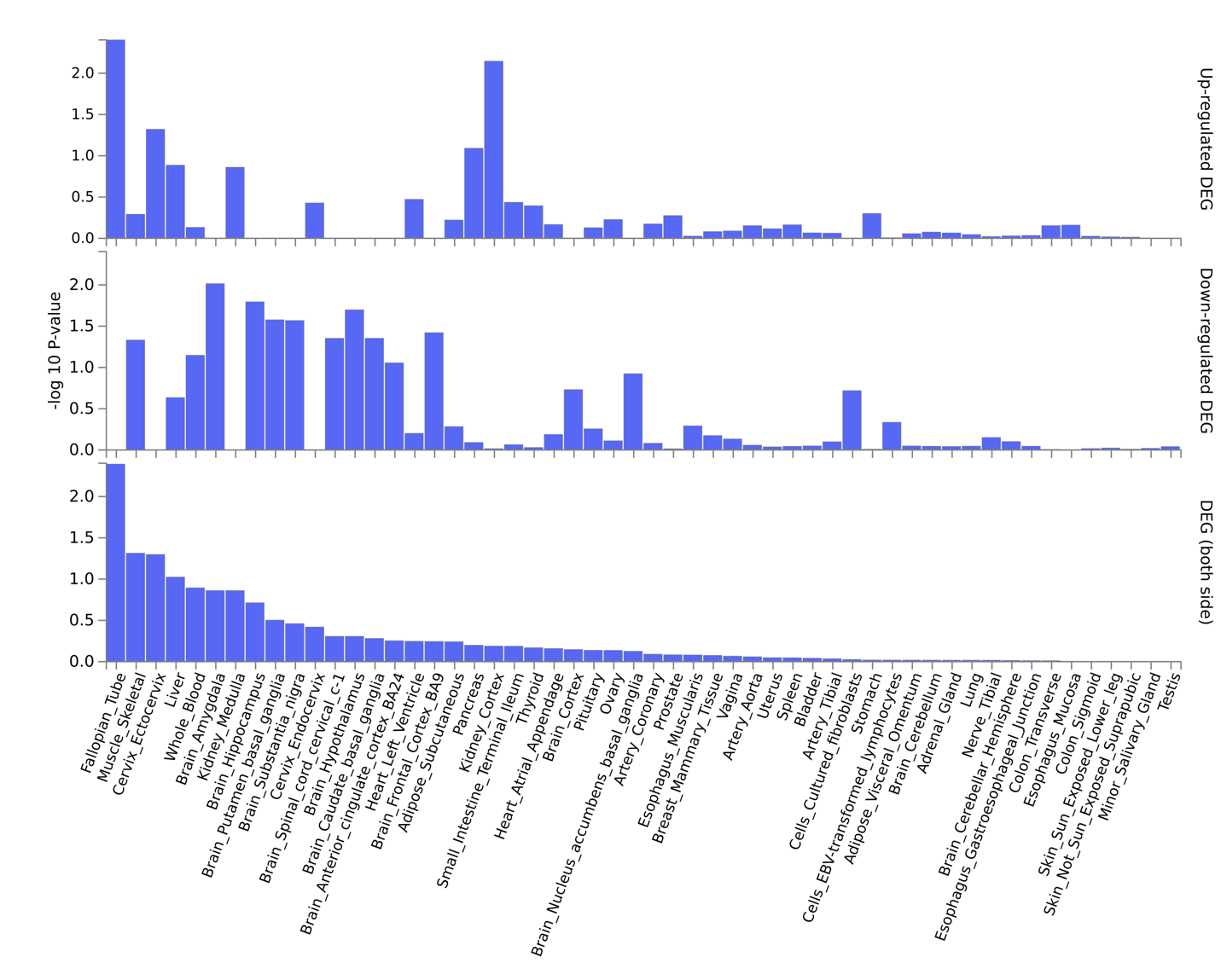

Supplement: Supplementary file 12 — Additional file 12. Supplementary Figures. [file 40246_2024_627_MOESM12_ESM.docx]
